# All‐Cause Acute Illness Hospitalisations in the Preceding Two Years Are Associated With Cognitive Decline in Older Adults: The Sydney Memory and Ageing Study

**DOI:** 10.1002/gps.70077

**Published:** 2025-05-01

**Authors:** Lucia Chinnappa‐Quinn, Ben C. P. Lam, Lara Harvey, Nicole A. Kochan, John D. Crawford, Steve R. Makkar, Henry Brodaty, Perminder S. Sachdev

**Affiliations:** ^1^ Centre for Healthy Brain and Ageing, Discipline of Psychiatry and Mental Health School of Clinical Medicine UNSW Sydney Australia; ^2^ Department of Anaesthesia Eastern Health Box Hill Australia; ^3^ School of Psychology and Public Health La Trobe University Melbourne Australia; ^4^ Falls, Balance and Injury Research Centre Neuroscience Research Australia Randwick Australia; ^5^ School of Population Health UNSW Sydney Australia; ^6^ Neuropsychiatric Institute Prince of Wales Hospital Randwick Australia

**Keywords:** aged, cognition, cognitive impairment, hospitalization, neuropsychological tests

## Abstract

**Objectives:**

Emerging evidence suggests all‐cause acute hospitalizations are associated with cognitive decline, rather than being associated only with specific inpatient contexts (surgery, critical care and delirium). This study clarifies this association in an Australian context.

**Methods:**

This study is a secondary analysis of four biennial waves of prospective population‐based neuropsychological measures from 1026 functionally independent Sydney Memory and Ageing Study participants aged 70–90 years at baseline, and contemporaneous probabilistically‐linked hospitalization data. The outcome measures were global cognition baseline (intercept) and change (slope) and their associations with hospitalization episodes and cumulative length of stay (cLOS) variables in five consecutive 2‐year time intervals.

**Results:**

One thousand twenty‐six individuals had a mean age of 78.8 years, a mean Mini‐Mental State Examination score of 28.7, a mean of 3.3 hospitalizations and 18.9 days in hospital over 10 years. Mean global cognition z‐score change/year was −0.133, adjusted for age, sex and education. Hospitalizations and cLOS in the final time interval were associated with a change in slope of −0.012 global cognition z‐score/hospitalization/year (Standard Error [SE] = 0.005, *p* = 0.014) and −0.002 z‐score/day‐in‐hospital/year (SE = 0.001, *p* < 0.001). Further investigation of these associations with time‐lagged models showed that pooled recent hospitalizations were associated with accelerated cognitive decline of −0.036 change in cognition/year/episode‐of‐hospitalization (SE = 0.012, *p* = 0.004) and −0.008 change in cognition/year/day‐in‐hospital (SE = 0.002, *p* < 0.001) rather than non‐recent hospitalizations (Wald test for difference between pooled recent and non‐recent effects had *p*‐values of 0.011 and < 0.001 for hospitalization episodes and days respectively).

**Conclusions:**

This study confirms and adds nuance to international findings that overnight hospitalization is associated with accelerated cognitive decline. This association was dose‐dependent, had a recency effect and was independent of illness severity in the case of cLOS. These findings suggest that all‐cause acute hospitalization may be a reversible risk factor for cognitive decline. This needs further clarification and the development of interventions to minimise the impact of acute illness hospitalization on cognitive trajectory. To this end, broadening the scope of acute care in the home and the prevention and treatment of neuroinflammation are priorities for further investigation.

**Summary**

- This paper confirms that all‐cause acute hospitalization is associated with accelerated cognitive decline in older Australian adults
- This association was greater for more recent hospitalizations suggesting the impact on cognition may recover with time
- Even non‐neurological acute illness was associated with cognitive decline, potentially via neuroinflammatory mechanisms
- Cumulative days in hospital were associated with worsened cognition even when illness severity was controlled for, suggesting the overnight stay itself contributes to worsened cognition

## 1 Background

Cognitive decline following acute illness hospitalization has been reported following surgery, critical care and admissions complicated by delirium [1]. However, recent evidence suggests that cognitive decline may follow any acute illness hospitalization (hereafter referred to as hospitalization) [2, 3, 4, 5, 6, 7].

Acute illness is characterised by systemic inflammation [8], in turn leading to neuroinflammation even with non‐neurological pathology, and may contribute to cognitive decline [8, 9]. Given the frequency of hospitalization in older adults [10], it may be an underestimated contributor to age‐related cognitive decline [11] and may interact with known cognitive risk factors [12].

The increase in longitudinal cognitive studies has facilitated the secondary analyses of neuropsychological data in relation to hospitalization [2, 3, 4, 5, 6, 7, 13], mostly investigating hospitalization as a binary exposure comparing hospitalized and non‐hospitalized groups. More recent studies have also investigated length of stay (LOS) variables and the effect of multiple hospitalizations [5, 7]. Most population‐based studies [2, 3, 4, 5, 13], and one meta‐analysis [14] reported evidence for worsened cognition following hospitalization. However, conclusions are limited since mainly categorical hospitalization variables are employed. In particular, the long‐term exposure to multiple serial hospitalizations makes the isolation of a non‐hospitalized comparison group challenging.

This study conducted a secondary analysis of longitudinal cognitive data from The Sydney Memory and Ageing Study combined with linked hospitalization records. The primary aim was to investigate the association of global cognition trajectory with the number and recency of hospitalization episodes and cumulative length of stay (cLOS). The methodological issue of multiple hospitalizations over time was addressed by using continuous hospitalization variables. The secondary aim was to identify potential interactions between hospitalization and known cognitive risk factors.

## 2 Materials and Methods

### 2.1 Participants

Participants were aged 70–90 years when recruited via electoral roll between September 2005 and November 2007 from metropolitan Sydney (Supporting Information S1: Table S1) and had a Mini‐Mental State Examination (MMSE) score ≥ 24 and no neurological or psychiatric comorbidities. Inclusion and exclusion criteria have been published (Supporting Information S1: Table S2) [15]. Ethics approval for the Sydney Memory and Ageing Study (1037 participants) and hospitalization data access (1026 of 1037 participants) was obtained (Supporting Information S1: p. 2).

### 2.2 Cognition Data

Four biennial waves of cognitive assessment using 10 neuropsychological tests (Supporting Information S1: Table S3) were standardized against the mean and standard deviation of the whole sample at Wave 1. Exploratory factor analysis with oblique rotation was used to group tests into approximate cognitive domains. Three factors had Eigen values greater than or close to 1 (4.57, 1.97 and 0.991 respectively) and explained 62% of the variance. This suggested three factors were underlying the cognitive tests, tapping language (Boston Naming Test, Semantic Fluency Animals, Controlled Oral Word Association Test), executive/spatial function (Trail Making Tests A and B, Digit Symbol Coding, Benton Visual Retention Test, Block Design) and memory (Logical Memory Story A delayed recall and Rey Auditory Verbal Learning Test). Factor‐based cognitive domain scores were then calculated by averaging the cognitive tests associated with each factor. The domain scores were then restandardized against the Wave 1 cognitive domain scores to provide z‐scores for Language, Memory and Executive/Spatial function for analysis (see below).

### 2.3 Hospitalization Data

Deidentified probabilistically‐linked records from July 1, 2001 to June 30 2014 were obtained from the New South Wales Admitted Patient Data Collection via the Centre for Health Record Linkage (Supporting Information S1: p. 56) [16]. The false positive rate for linkage for this sample was 0.5%. Of the 1026 participants, 1016 (99%) were able to be linked to hospital records in New South Wales, seven of these having no NSW hospitalizations. Ten were unable to be linked (Supporting Information S1: p. 63). Non‐psychiatric overnight hospitalizations were identified as the exposure of interest.

The hospitalization data used were from a 10‐year time period preceding the fourth (final) cognitive assessment for each individual. These hospitalizations were grouped into five consecutive approximately 2‐year time intervals according to the timing of biennial cognitive assessments for each participant: four intervals preceding each cognitive assessment wave (pre‐wave intervals 1 to 4) and one lookback 2‐year interval beginning 4 years prior to and ending 2 years prior to the first cognitive assessment (interval 0) (see Supporting Information S1: Figure S1 for an example). A 4‐year lookback was used for hospitalizations given evidence from previous studies of long‐term associations with cognitive changes [3, 4].

The hospitalization variables used were number of hospitalization episodes and number of days stayed in hospital (i.e., cumulative length‐of‐stay or cLOS) within each time interval. The cLOS variable was defined as the total number of days in hospital in that time interval (i.e., two hospitalizations for two and 4 days respectively within one time interval would constitute a cLOS of 6 days) (Supporting Information S1: Figure S1). This variable was added to increase sensitivity in quantifying the association of hospitalization and cognition, given that greater illness severity likely requires longer LOS.

Hospitalizations with primary central nervous system (CNS) diagnoses potentially contributory to cognitive decline were identified by Australian Refined Diagnosis‐Related Groups codes [17]. The likely confounding effects on cognition of these was investigated by conducting a sensitivity analysis removing these hospitalizations.

### 2.4 Covariates

Participant comorbidities were available from both self‐report Sydney Memory and Ageing Study and linked hospitalization data. Self‐reported comorbidities included more specific and longitudinal information and thus, were used for adjustment of cognitive outcomes. In contrast, coded comorbidities in the hospital linked data were more likely to provide a snapshot of conditions requiring pharmacological management as an inpatient or influencing the complexity and cost of the hospitalization.

Potentially confounding demographic and comorbidity variables were selected with reference to similar studies [2, 3, 4] and known risk factors for older age cognitive decline [12], and prepared from baseline self‐report data (Supporting Information S1: p. 35) and genetic testing for the Apolipoprotein E ε4 allele (APOE*4). Demographics included age, sex, education and Non‐English speaking background (NESB) (likely to disadvantage participants completing tests developed for native English speakers [18]). Comorbidities included neurological, vascular, cardiac, respiratory and thyroid disease, anaemia, diabetes mellitus, cancer, smoking pack‐years, alcohol consumption, body mass index, self‐reported general health, Geriatric Depression Scale (GDS) score [19], and APOE*4 carrier status.

The Charlson Comorbidity Index with 1‐year lookback (CCI) [20] was calculated for each hospitalization from diagnoses provided in the linked data. In the absence of specific illness severity data such as inflammatory markers and clinical observations to further nuance the degree of physiological insult from the hospitalization, CCI was used in separate models as a surrogate marker of acute illness severity [21]. Maximum CCI for an individual in each time interval was used for analysis. CCI was added to interactions models as both an estimate of comorbidity burden and illness severity.

### 2.5 Statistical Analysis

Hospitalization data were prepared using SAS Enterprise Guide v7.2 (SAS Institute, Cary, NC, USA) and then merged Sydney Memory and Ageing Study data and prepared in SPSS v26 (IBM SPSS Statistics for Windows, Version 26.0. IBM Corp). MPlus v8 was used for structural equation modelling (MPlus Diagrammer. Version 1.6 (1). 2012–2018).

Longitudinal confirmatory factor analysis was conducted on the cognitive domain scores to estimate global cognition at each time point. Measurement invariance of global cognition was examined over time (Supporting Information S1: Figure S2). Latent growth curve modelling (specifically a Curve‐of‐Factors model [CFM]) was then used to estimate the primary cognitive outcome measures: latent global cognition intercept (estimated baseline level of cognition) and latent global cognition slope (estimated linear rate of change in cognition per year) (Supporting Information S1: Figure S3) [22].

**FIGURE 1:**
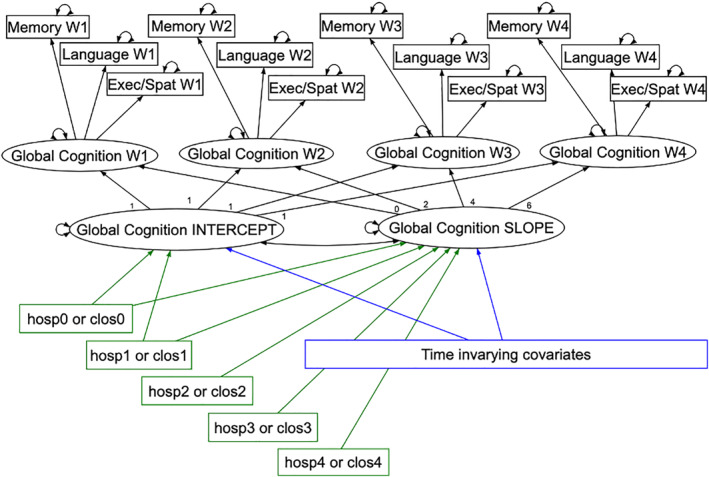
Curve‐of‐factors model to estimate the intercept and slope of global cognition from latent global cognition at waves 1–4 (W1–W4), in turn estimated from three cognitive domain measures, memory, language and executive/visuospatial (exec/spat) function, at each wave (W1–W4) (black arrows), and hospitalization predictors in five time intervals (hosp0–hosp4: hospitalizations episodes or clos0–los4: cumulative acute hospitalization length of stay in days) (green arrows) and time‐invarying covariates (drawn with http://semdiag.psychstat.org/) (blue arrows). The curved arrows represent residual variances or measurement error of latent factors or measured variables respectively.

Covariates were tested individually and included in the final models if they were associated with intercept and/or slope at *p* < 0.10. Finally, hospitalization variables were added to the models (Figure 1).

**FIGURE 2:**
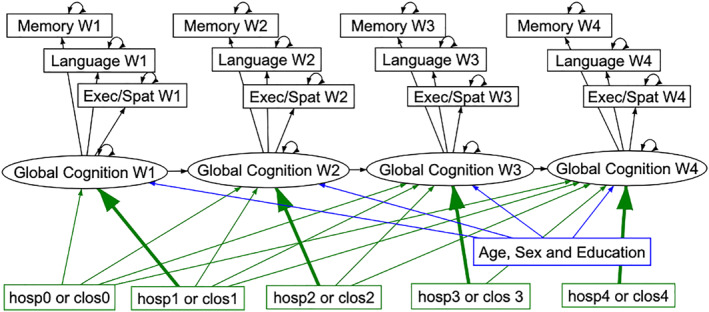
Lagged model using the LCFA (Supporting Information S1: Figure S2) to estimate global cognition at each time point with five‐interval hospitalization predictors adjusted for age, sex and education, with recent hospitalization predictors (bold arrows) and non‐recent hospitalization predictors (lighter green arrows) respectively (i.e., for w4 cognition the hosp0–3 intervals), constrained to be equal.

Statistical significance was set at a *p*‐value of < 0.05 for primary outcome and interaction effects. Model fit was evaluated using the comparative fit index (CFI), root mean square error of approximation (RMSEA), and standardized root mean square residual (SRMR). Interaction effects of age, education, sex and APOE*4 variables, as well as CCI, with hospitalization variables were investigated in exploratory fashion, and analysed to ascertain strength and direction. Models were estimated using maximum likelihood with robust standard errors to account for potential non‐normal distributions. Missing values were managed by full information maximum likelihood for cognitive outcomes and multiple imputation for covariates [23]. Post‐hoc time‐lagged models were conducted to clarify the effects of recent and non‐recent hospitalization intervals on latent global cognition at each wave (Figure 2 and Supporting Information S1: Figure S4).

## 3 Results

### 3.1 Demographic, Cognition and Hospitalization Characteristics

At baseline, the sample means included an age of 78.8 years, 11.6 education years and an MMSE score of 28.7. Over 95% had no impairment of instrumental activities of daily living (Bayer‐Activities of Daily Living Scale [24] < 3) and 15.9% were from a NESB. Cancer (47.3%), vascular disease (34.2%) including ischaemic heart disease, and other cardiac disease including arrhythmia and valvular heart disease (16.6%) were the most common self‐reported comorbidities (Table 1 and Supporting Information S1: Table S1). Baseline characteristics were similar to Australian population data [25, 26], except for a higher proportion of participants being tertiary educated and living independently.

**TABLE 1:** Demographic and self‐report comorbidity descriptives at wave 1 (*n* = 1026).

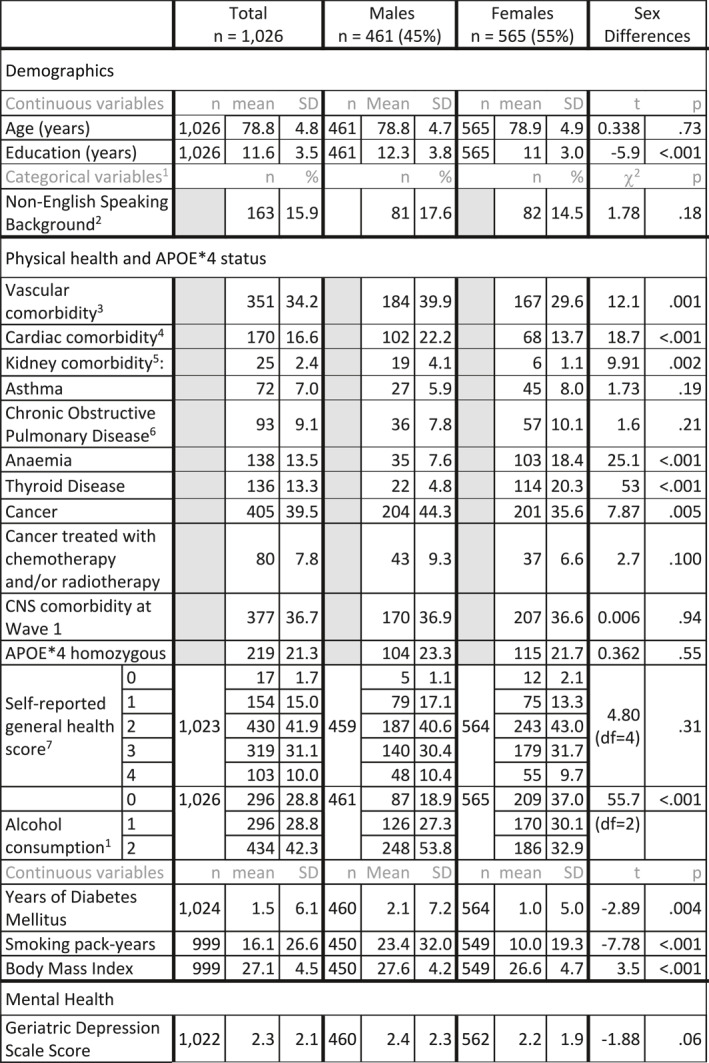

^a^ With the exception of Alcohol consumption and Self‐rated health, categorical variables are binary denoting presence or absence of the condition.

^b^ Individuals were included only if they had adequate English to complete a psychometric assessment (Supporting Information S1: Table S2).

^c^ Acute myocardial infarct, angina, aortic aneurysm or claudication.

^d^ Atrial fibrillation, other arrhythmias, cardiomyopathy or valvular heart disease.

^e^ Kidney disease or dialysis in the past.

^f^ Emphysema, chronic bronchitis or bronchiectasis.

^g^ 0: poor, 1: fair, 2: good, 3: very good, 4: excellent.

^h^ 0: None or < monthly in the last year, 1: 2–4 times per month or 2–3 times per week, 2: 4–6 times per week or daily.

Available neuropsychological data and cognitive domain scores across waves are presented in Supporting Information S1: Figure S5 and Table S5 respectively. Longitudinal confirmatory factor analysis was conducted to test measurement invariance over time, and strong measurement invariance was established (Supporting Information S1: Table S6). In the CFM, the estimated mean global cognition slope was −0.105 z‐score change/year (Standard Error [SE] = 0.007, *p* < 0.001, Supporting Information S1: Table S7), and adjusted for age, sex and education, was −0.133 z‐score change/year (SE = 0.009, *p* < 0.001). A quadratic slope did not improve model fit and was not further considered.

Regarding hospitalizations, in the approximately 10‐year follow‐up from 2001 to the fourth cognitive assessment, there were 3352 overnight hospitalizations, of which 1479 (44.1%) were emergency and 142 (4.2%) included critical care (Supporting Information S1: Table S8). Most participants (82.3%) were hospitalized at least once with a mean of 3.3 hospitalizations and 18.9 days in hospital. A few individuals had several hospitalizations: the maxima being 31 hospitalizations and 257 days in hospital. In each 2‐year interval, mean hospitalizations ranged from 0.55 to 0.93 episodes, and cLOS from 3.0 to 5.7 days (Supporting Information S1: Table S9). There were 432 ICD‐10 codes recorded for primary admission diagnoses, mostly being cardiovascular disease, osteoarthritis, atrial fibrillation and chronic obstructive pulmonary disease (Supporting Information S1: Table S10), and 3.7% (124 of 3352) were for CNS diagnoses (Supporting Information S1: Table S11).

### 3.2 Hospitalization and Cognition Intercept and Slope

The hospitalization effects in the final CFM were adjusted firstly for age, sex and education (Supporting Information S1: Tables S12 and S13), and then the selected covariates (NESB, years of diabetes, GDS score, smoking packyears, alcohol consumption and self‐reported general health and APOE*4 allele (Supporting Information S1: Tables S14 and S15). All model fit indices were adequate.

Estimated baseline global cognition z‐score (latent cognition intercept) was not predicted by number of hospitalization episodes or cLOS. Estimated change in global cognition z‐score (latent cognition slope) was predicted by the number of hospitalization episodes in the pre‐Wave 4 interval (i.e., the 2 years before Wave 4) (*b* = −0.012, SE = 0.005, *p* = 0.014) (Table 2), and cLOS in the pre‐Wave 4 interval (*b* = −0.002, SE = 0.001, *p* < 0.001) (Table 3). Effects were similar for sensitivity analyses with CNS hospitalizations removed (Supporting Information S1: Tables S16 and S17).

**TABLE 2:** The association of the number of hospitalizations in five time intervals with global cognition intercept and slope, adjusted for selected covariates.

	Adjusted for age, sex, education and comorbidities model fit: CFI = 0.954, RMSEA = 0.041, SRMR = 0.065^a^; R‐squared for intercept = 0.449, slope = 0.145							
	Estimated effect on cognition intercept				Estimated effect on cognition slope			
	b^b^	SE	*p*	*β* ^c^	b	SE	*p*	*β*
Interval 0 (hosp0)	−0.034	0.025	0.18	−0.041	−0.002	0.006	0.70	−0.025
Pre‐wave 1 (hosp1)	0.034	0.042	0.42	0.025	0.003	0.008	0.76	0.017
Pre‐wave 2 (hosp2)					0.003	0.009	0.77	−0.020
Pre‐wave 3 (hosp3)					−0.006	0.008	0.42	−0.057
Pre‐wave 4 (hosp4)					**−0.012**	**0.005**	**0.014**	**−0.121**
Age	**−0.107**	**0.010**	**<** **0.001**	**−0.382**	**−0.010**	**0.002**	**<** **0.001**	**−0.312**
Female	**0.311**	**0.097**	**0.001**	**0.115**	0.005	0.015	0.75	0.016
Education	**0.141**	**0.015**	**<** **0.001**	**0.365**	−0.002	0.002	0.33	−0.048
Non‐English speaking background	**−0.935**	**0.130**	**<** **0.001**	**−0.254**	0.010	0.019	0.59	0.026
Cancer	0.139	0.084	0.10	0.051	0.013	0.014	0.34	0.044
Geriatric depression scale score	**−0.064**	**0.022**	**0.003**	**−0.099**	0.003	0.004	0.45	0.038
APOE*4 allele	**−0.251**	**0.103**	**0.015**	**−0.078**	**−0.050**	**0.018**	**0.005**	**−0.141**
Years of diabetes mellitus	**−0.010**	**0.005**	**0.048**	**−0.046**	−0.001	0.001	0.53	−0.036
Smoking Pack‐years	**0.005**	**0.002**	**0.005**	**0.090**	0.000	0.000	0.19	0.059
Alcohol consumption	0.064	0.051	0.21	0.039	0.005	0.009	0.59	0.026
Self‐reported general health	0.093	0.050	0.06	0.063	0.008	0.008	0.37	0.046

*Note:* The bold values are the effect sizes which had a *p*‐value of < 0.05.

^a^ CFI: comparative fit index, RMSEA: Root Mean Square Error of Approximation, SRMR: Standardized Root Mean Square Residual.

^b^ The unstandardized effect estimate ‘b’ reported in Tables 2 and 3 refers to change in cognition in *z*‐score units for every unit change in the predictor (hospitalization or covariate variable), and thus the magnitude and direction of the association of the predictor with cognition intercept or slope. Within each model, the ‘b’ for cognition intercept is given on the left and the ‘b’ for slope on the right.

^c^ The standardized effect estimate.

**TABLE 3:** The association of cumulative length of stay (cLOS) in five time intervals with global cognition intercept and slope, adjusted for selected covariates.

	Adjusted for age, sex, education and comorbidities Model fit: CFI = 0.953, RMSEA = 0.041, SRMR = 0.061^a^; R‐squared for intercept = 0.446, slope = 0.172							
	Estimated effect on cognition intercept				Estimated effect on cognition slope			
	b^b^	SE	*p*	*β* ^c^	b	SE	*p*	*β*
cLOS interval 0 (clos0)	−0.001	0.003	0.74	−0.011	0.000	0.001	0.60	0.037
cLOS pre‐Wave 1 (clos1)	−0.006	0.005	0.23	−0.038	0.002	0.001	0.14	0.098
cLOS pre‐Wave 2 (clos2)					−0.001	0.001	0.20	−0.097
cLOS pre‐Wave 3 (clos3)					−0.001	0.001	0.42	−0.053
cLOS pre‐Wave 4 (clos4)					**−0.002**	**0.001**	**<** **0.001**	**−0.185**
Age	**−0.107**	**0.010**	**<** **0.001**	**−0.383**	**−0.009**	**0.002**	**<** **0.001**	**−0.299**
Female	**0.315**	**0.097**	**0.001**	**0.117**	0.007	0.015	0.65	−0.022
Education	**0.142**	**0.015**	**<** **0.001**	**0.366**	−0.002	0.002	0.30	−0.050
Non‐English speaking background	**−0.934**	**0.130**	**<** **0.001**	**−0.254**	0.008	0.019	0.68	0.019
Cancer	0.143	0.084	0.09	0.052	0.011	0.014	0.42	0.037
Geriatric depression scale score	**−0.060**	**0.022**	**0.005**	**−0.093**	0.003	0.004	0.45	0.037
APOE*4 allele	**−0.253**	**0.104**	**0.015**	**−0.078**	**−0.054**	**0.018**	**0.002**	**−0.149**
Years of diabetes mellitus	**−0.010**	**0.005**	**0.049**	**−0.046**	−0.001	0.001	0.59	−0.030
Smoking Pack‐years	**0.005**	**0.002**	**0.004**	**0.092**	0.000	0.000	0.32	0.043
Alcohol consumption	0.058	0.051	0.26	0.036	0.007	0.009	0.43	0.038
Self‐reported general health	0.086	0.050	0.08	0.058	0.008	0.008	0.35	0.047

*Note:* The bold values are the effect sizes which had a *p*‐value of < 0.05.

^a^ CFI: comparative fit index, RMSEA: Root Mean Square Error of Approximation, SRMR: Standardized Root Mean Square Residual.

^b^ The unstandardized effect estimate ‘b’ reported in Tables 2 and 3 refers to change in cognition in *z*‐score units for every unit change in the predictor (hospitalization or covariate variable), and thus the magnitude and direction of the association of the predictor with cognition intercept or slope. Within each model, the ‘b’ for cognition intercept is given on the left and the ‘b’ for slope on the right.

^c^ The standardized effect estimate.

Thus cognitive slope over 6 years was predicted by the hospitalizations in the 2 years prior to the fourth assessment, but not earlier hospitalizations. To further understand these associations between the pre‐Wave 4 interval hospitalization predictors and cognitive slope, post‐hoc time‐lagged models were tested to investigate the effects of recent and non‐recent hospitalizations for each interval. The effects of recent hospitalizations trended towards significance (pre‐waves 3 and 4 and Waves 3 and 4 cognition respectively) or were significant (pre‐wave 2 and Wave 2 cognition) (Supporting Information S1: Tables S18 and S19). Thus, to overcome the issue of low power due to the low variance of individual hospitalization predictors, recent and non‐recent effects were fixed to be equal separately allowing recent and non‐recent effects to be pooled (6) (Figure 2). Pooled recent hospitalization effects were associated with accelerated cognitive decline of −0.036 change in cognition/year/episode‐of‐hospitalization (SE = 0.012, *p* = 0.004), and −0.008 change in cognition/year/day‐in‐hospital (SE = 0.002, *p* < 0.001). Non‐recent hospitalization effects were either not associated or associated with improvement in cognitive slope (episodes: *b* = 0.004, SE = 0.006, *p* = 0.467; cLOS: *b* = 0.002, SE = 0.001, *p* = 0.007). Wald test for difference between pooled recent and non‐recent effects was significant for hospitalizations (*X* ^2^ = 6.542, df = 1, *p* = 0.011) and cLOS (*X* ^2^ = 20.355, df = 1, *p* < 0.001) (Table 4).

**TABLE 4:** Time‐lagged model with fixed effects: showing the association of number of hospitalizations and cumulative length of stay (cLOS) in five time intervals with the most recent interval and earlier intervals fixed to be equal separately, on global cognition at each time point adjusted for age, sex and education, and the difference between recent and non‐recent effects compared.

Estimated effects on global cognition at each timepoint (GCOG1‐GCOG4) adjusted for age, sex, education^a^			
	b	SE	*p*
Number of hospitalizations			
Fixed effect for hospitalizations in earlier time interval(s)^b^	0.004	0.006	0.47
Fixed effect for hospitalizations in the most recent time interval^c^	**−0.036**	**0.012**	**0.004**
Model fit: CFI = 0.976, RMSEA = 0.041, SRMR = 0.043; R‐squared GCOG1 = 0.342, GCOG2 = 0.937, GCOG3 = 0.917, GCOG4 = 0.946			
Wald test for difference between recent and earlier effects *χ* ^2^ = 6.542, df = 1, *p*‐value = 0.011			
Cumulative length of stay			
Fixed effect for cLOS in earlier time interval(s)^b^	**0.002**	**0.001**	**0.007**
Fixed effect for cLOS in the most recent time interval^c^	**−0.008**	**0.002**	**<** **0.001**
Model fit: CFI = 0.976, RMSEA = 0.042, SRMR = 0.043; R‐squared GCOG1 = 0.344, GCOG2 = 0.940, GCOG3 = 0.917, GCOG4 = 0.948			
Wald test for difference between recent and earlier effects ***χ*** ^**2**^ **= 20.355, df = 1, *p*‐value** **< 0.001**			

*Note:* The bold values are the effect sizes which had a *p*‐value of < 0.05.

^a^ Age, sex and education effects were similar to previous models and are not shown in the table.

^b^ Pooled effects of intervals 0 hospitalizations on Wave 1 cognition, intervals 0 and pre‐Wave 1 hospitalizations on Wave 2 cognition, intervals 0, pre‐Wave 1 and pre‐Wave 2 hospitalizations on Wave 3 cognition and intervals 0, pre‐Wave 1, pre‐Wave 2 and pre‐Wave 3 hospitalizations on Wave 4 cognition (i.e., hospitalizations earlier than 2 years preceding cognition assessment).

^c^ Pooled effects of pre‐Wave 1 interval hospitalizations on Wave 1 cognition, pre‐Wave 2 interval hospitalizations on Wave 2 cognition, pre‐Wave 3 interval hospitalizations on Wave 3 cognition and pre‐Wave 4 interval hospitalizations on Wave 4 cognition (i.e., most recent hospitalizations within 2 years preceding cognition assessment).

Regarding the covariates, older age and APOE*4 allele were strongly associated with lower baseline cognition and greater cognitive decline. Conversely, female sex and higher education years were associated with higher baseline cognition. Covariate associations are given in Tables 2 and 3.

Interactions between hospitalization variables and age, sex, education and APOE*4 were explored together (Supporting Information S1: Tables S20 and S21). Only hospitalization episodes in the pre‐Wave 2 interval interacted with age, with hospitalizations accelerating cognitive decline more in younger individuals and improving cognition in older individuals. All other interaction terms with hospitalization episodes and cLOS variables were not significant.

In separate analyses adjusting for CCI, age, sex and education, hospitalization episodes no longer predicted cognition in the CFM or time‐lagged models (Supporting Information S1: Tables S22 and S24). However, cLOS remained significant (Supporting Information S1: Tables S23 and S25). To understand the separate contribution of CCI to cognitive decline, models were also run with CCI, age, sex and education, without hospitalization predictors. In the CFM, CCI in the pre‐Wave 4 interval was associated with latent cognitive slope (*b* = −0.013, SE = 0.004, *p* = 0.003), and in the lagged model, in the pre‐wave 2 interval with Wave 2 latent global cognition (*b* = −0.051, SE = 0.026, *p* = 0.046) (Supporting Information S1: Tables S26 and S27). There was a small magnitude interaction of hospitalization and CCI in the pre‐wave 4 interval, but not with cLOS (Supporting Information S1: Tables S28 and S29).

## 4 Discussion

This study investigated the association of a 6‐year cognitive trajectory in functionally independent older Australians, with 10 years of prior and contemporaneous hospitalizations, using latent growth modelling.

Regarding sample characteristics, the proportion of participants hospitalized was comparable to or higher than similar studies [2, 3, 4]. CNS hospitalization rates were comparable to Australian rates (3.7% vs. 4.6% respectively) [17]. Regarding LOS, in this sample, mean LOS per hospitalization was 5.8 days. Thus, the mean estimated effect of a pre‐Wave 4 hospitalization episode of −0.012 is consistent with the mean estimated effect of −0.002 per day of cLOS. Furthermore, this was similar to the Australian mean LOS in over 70‐year‐old adults per hospitalization of 5.6 days [27]. Changes in memory and language (Supporting Information S1: Table S5) were consistent with a similar Australian study [28] and unadjusted estimated mean global cognition slope was comparable to the Rush Memory and Ageing Project sample (−0.109 z‐score units/year, SE = 0.004, *p* < 0.001) [11]. Thus the results are generalisable to the Australian population and to other similar healthcare systems.

This is the first study, to the authors' knowledge, examining hospitalization associations with cognition temporally. Time‐lagged models in these analyses show the association of more recent (in the preceding 2 years) hospitalizations with cognitive decline as compared to earlier hospitalizations. This suggests decline associated with hospitalization may resolve, at least in part, with time.

Regarding the magnitude of this association, pre‐Wave 4 hospitalizations were associated with comparable acceleration in cognitive decline (−0.012 z‐score/hospitalization/year SE = 0.005 *p* = 0.014) to the effect estimate of 1 year of increased age on cognitive slope (−0.010 z‐score/hospitalization/year SE = 0.002 *p* < 0.001) (Table 2). Additionally pooled recent hospitalizations were associated with a significantly lower cognition at the next cognitive assessment for all assessment waves (−0.036 z‐score/hospitalization SE = 0.012 *p* = 0.004) (Table 4). The relationship of cognition and function is a complex one and thus the clinical significance of this change is difficult to pinpoint. More research is needed to specifically clarify the relationship of global cognition and function. Nevertheless, there is much preliminary evidence for associations between instrumental activities of daily living (ADLs) and subtle cognitive changes. In the Sydney Memory and Ageing study sample, there was strong correlation between cognitive domain decline and impaired high cognitive demand ADLs [29]. Subtle cognitive changes in Rush Memory and Ageing project participants have been associated with impaired decision‐making, vulnerability to scams, worse financial and health literacy, increased risk aversion and temporal discounting (i.e., the inability to make decisions with delayed advantage) [30, 31, 32, 33]. Changes in global cognition slope following hospitalization in this sample, particularly with repeated hospitalization, may well contribute to similar functional changes.

With regards to covariates, associations with global cognition intercept and slope were similar to those previously reported for this sample in relation to MCI [34], and known risk factors for dementia [12]. CNS comorbidity at baseline was not associated with cognition, likely due to stringent inclusion criteria.

CCI was moderately correlated with hospitalization episodes and cLOS (Supporting Information S1: Figure S6), consistent with patients with greater comorbidity and illness severity requiring frequent and lengthier hospitalizations. Notably, adjustment for CCI removed the unique predictive value of hospitalization episodes for cognition, but not cLOS. Furthermore, both CCI and hospitalization episodes were associated with accelerated cognitive decline in separate models, but not in the same model, suggesting shared predictive value. Furthermore, the unique predictive value of cLOS after adjustment for illness severity suggests that duration of hospitalization itself, as distinct from the acute illness, is associated with worse cognition. The mechanism of this may be linked to increased cLOS contributing to a vicious cycle of increased delirium risk, which in turn further increases LOS [35, 36].

Many factors associated with hospitalization may contribute to cognitive decline. These include metabolic derangement, social isolation, pain, exaggerated or inadequate stress response, sensory and sleep deprivation, and polypharmacy, all of which have been implicated in delirium [37], but likely contribute in the absence of delirium also [14], possibly via neuroinflammatory mechanisms [8, 9]. Findings from this analysis give weight to a neuroinflammatory aetiology. Firstly, higher CCI as a surrogate marker of greater illness severity is likely associated with higher levels of inflammation, and in this analysis, had shared prediction of accelerated cognitive decline with hospitalizations. Illness severity, rather than comorbid burden, is likely responsible for the association with CCI, given the analyses adjusted for self‐report comorbidities did not show similar shared predictive value. Secondly, the recency effect suggests a transient aspect to the aetiology, potentially consistent with the resolution of an inflammatory process. These associations remained independent of CNS comorbidity and CNS hospitalizations suggesting that all‐cause hospitalization may be a reversible neuroinflammatory aetiological factor for cognitive decline.

These findings suggest hospitalizations are an underestimated contributor to older age cognitive decline. Addressing aetiological factors for this accelerated cognitive decline could tangibly improve cognition, longevity of employment and quality of life [38, 39]. This may in turn lead to decreased risk of dementia and residential care requirement, improved life expectancy and favourable health economic outcomes [39, 40, 41].

Thus, further investigation is mandatory to elucidate the complex interrelationship of hospitalization, illness severity, comorbidities and cognition and their clinical and public health implications. Future research should incorporate as standard, frequent cognitive measures and contemporaneous hospitalization data including alternative LOS variables, types of hospitalization, inflammatory markers and inpatient complications, in particular delirium. Neuroinflammatory mechanisms are being studied with increasing interest [8] and anti‐neuroinflammatory therapies showing promise in the perioperative context [42] may be relevant to all‐cause acute illness. Innovative models have been investigated in randomised studies comparing hospital versus home acute care [43, 44, 45]. These studies show that home‐care models could improve cognitive and functional outcomes, clinical complication rates, patient satisfaction and cost, potentially revolutionising acute care delivery.

Strengths of this study are as follows. The neuropsychological test battery was designed to be sensitive for the diagnosis of predementia syndromes [15]. The cLOS variable allowed further elucidation of the role of LOS in hospitalization exposure. In this sample, hospitalization was associated with rate of decline rather than baseline cognition reinforcing the need for cognition to be investigated as a trajectory [46]. To that end, this study shows that latent growth modelling in a structural equation modelling framework is a useful technique in longitudinal cognitive research, facilitating the modelling of multiple predictors and outcomes simultaneously. Additionally, the use of latent factors is advantageous to account for different weightings and residual variances of factor scores [47]. Comprehensive low‐cost hospitalization data access without survival and respondent bias, was facilitated by prior consent to healthcare data collection and a reliable linkage process. Evidence supports the accuracy of linked health records being similar to medical records and superior to self‐reported hospitalization data [48, 49].

### 4.1 Limitations

Longitudinal studies with long time periods between cognitive assessments [2, 3, 4, 6] are hampered by an early follow‐up blind spot. In this analysis, only 10% of cognitive assessments occurred within 4 months of a hospitalization. The sample had high mean education years potentially leading to underestimation of the hospitalization associations for the population. Additionally, linked health records are limited by potential inaccurate data coding [50] and the absence of inpatient data, such as inflammatory markers, oxygenation and admission complications (Supporting Information S1: p. 56). Conclusions regarding LOS are limited by analysing LOS by time interval, making it impossible to differentiate several short hospitalizations from few lengthy hospitalizations within the same time interval.

### 4.2 Conclusion

In summary, all‐cause hospitalizations in the preceding 2 years were associated with accelerated cognitive decline, even in functionally independent older Australians with minimal pre‐existing neurological comorbidity. These Australian findings support and add nuance to the growing global literature on the association of hospitalization and cognition, raising more questions for further investigation [2, 3, 4, 5, 6].

## Ethics Statement

Ethics approval for the Sydney Memory and Ageing Study was obtained from the University of New South Wales and South Eastern Sydney and Illawarra Area Health Service Ethics Committees (approval numbers 2015‐20 HC14327 and 2020‐25 HC190962). Access to linked New South Wales (NSW) Health data was approved by the NSW Population & Health Services Research Ethics Committee (AU RED Reference HREC/15/CIPHS/11 up to 2025) for 1, 026 of the 1, 037 (98.9%) individuals, on the basis that they had given consent for Medicare records to be accessed.

## Consent

Written informed consent was obtained from participants. Each participant was free to refuse a specific part of the assessment (such as blood sampling or MRI). Formal feedback from assessments was provided in written reports to the participants and their general practitioners. If tests revealed that the participant required immediate medical or psychiatric attention, they were contacted by telephone as was their general practitioner [15].

## Conflicts of Interest

H.B. is or has been an advisory board member or consultant to Biogen, Eisai, Eli Lilly, Roche and Skin2Neuron, as well as a member of the Clinical Advisory Boards for Cranbrook Care and Montefiore Homes. P.S. was on the Advisory Committees for Biogen and Roche from 2020 to 2022 and was paid an honorarium for a lecture by Alkem Laboratories in 2023. All other authors do not have conflicts of interest.

## Supporting information

Supporting Information S1

## Data Availability

The de‐identified data we analysed are not publicly available, but requests to the corresponding author for the data will be considered on a case‐by‐case basis. All authors had full access to all the data (including statistical reports and tables) related to the study.

## References

[1] L. Chinnappa‐Quinn , M. Bennett , S. R. Makkar , N. A. Kochan , J. D. Crawford , and P. S. Sachdev , “Is Hospitalisation a Risk Factor for Cognitive Decline in the Elderly?,” Current Opinion in Psychiatry 33, no. 2 (2019): 170–177, 10.1097/yco.0000000000000565. 31652137

[2] W. J. Ehlenbach , C. L. Hough , P. K. Crane , S. J. P. A. Haneuse , S. S. Carson , and J. R. Curtis , “Association Between Acute Care and Critical Illness Hospitalization and Cognitive Function in Older Adults. Research Support, N.I.H., Extramural Research Support, Non‐U.S. Gov't Research Support, U.S. Gov't, Non‐P.H.S,” JAMA 303, no. 8 (February 2010): 763–770. 20179286 10.1001/jama.2010.167 PMC2943865

[3] R. S. P. Wilson , L. E. S. Hebert , P. A. S. P. Scherr , X. M. Dong , S. Leurgens , and D. Evans , “Cognitive Decline After Hospitalization in a Community Population of Older Persons,” Neurology 78, no. 13 (2012): 950–956, 10.1212/wnl.0b013e31824d5894. 22442434 PMC3310309

[4] C. H. Brown , S. Armdp , C. J. Jmdpmhs , et al., “Association of Hospitalization With Long‐Term Cognitive and Brain MRI Changes in the ARIC Cohort,” Neurology 84, no. 14 (2015): 1443–1453, 10.1212/wnl.0000000000001439. 25762715 PMC4395884

[5] J. Sprung , D. S. Knopman , R. C. Petersen , et al., “Association of Hospitalization With Long‐Term Cognitive Trajectories in Older Adults,” Journal of the American Geriatrics Society 69, no. 3 (October 2020): 660–668, 10.1111/jgs.16909. 33128387 PMC7969446

[6] J. Hallgren , E. I. Fransson , C. A. Reynolds , D. Finkel , N. L. Pedersen , and A. K. Dahl Aslan , “Cognitive Trajectories in Relation to Hospitalization Among Older Swedish Adults,” Archives of Gerontology and Geriatrics 74 (January 2018): 9–14, 10.1016/j.archger.2017.09.002. 28923532

[7] D. Park , H. S. Kim , and J. H. Kim , “The Effect of All‐Cause Hospitalization on Cognitive Decline in Older Adults: A Longitudinal Study Using Databases of the National Health Insurance Service and the Memory Clinics of a Self‐Run Hospital,” BMC Geriatrics 23, no. 1 (February 2023): 61, 10.1186/s12877-022-03701-4. 36721117 PMC9890792

[8] Y. Sun , Y. Koyama , and S. Shimada , “Inflammation From Peripheral Organs to the Brain: How Does Systemic Inflammation Cause Neuroinflammation?,” Frontiers in Aging Neuroscience 14 (2022): 903455, 10.3389/fnagi.2022.903455. 35783147 PMC9244793

[9] S. Subramaniyan and N. Terrando , “Neuroinflammation and Perioperative Neurocognitive Disorders,” Anesthesia & Analgesia 128, no. 4 (April 2019): 781–788, 10.1213/ANE.0000000000004053. 30883423 PMC6437083

[10] AIHW AIoHaW . Admitted Patient Care 2015‐16: Australian Hospital Statistics (Australian Institute of Health and Welfare, 2017).

[11] P. A. Boyle , R. S. Wilson , L. Yu , et al., “Much of Late Life Cognitive Decline Is Not Due to Common Neurodegenerative Pathologies,” Annals of Neurology 74, no. 3 (September 2013): 478–489, 10.1002/ana.23964. 23798485 PMC3845973

[12] S. Gauthier , C. Webster , S. Servaes , J. A. Morais , P. Rosa‐Neto . World Alzheimer Report 2022: Life After Diagnosis: Navigating Treatment, Care and Support (Alzheimer’s Disease International, 2022).

[13] B. D. James , R. S. Wilson , A. W. Capuano , et al., “Hospitalization, Alzheimer's Disease and Related Neuropathologies, and Cognitive Decline,” Annals of Neurology 86, no. 6 (December 2019): 844–852, 10.1002/ana.25621. 31614018 PMC6973140

[14] L. Chinnappa‐Quinn , S. R. Makkar , M. Bennett , et al., “Is Hospitalization a Risk Factor for Cognitive Decline in Older Age Adults?,” International Psychogeriatrics 34, no. 11 (2020): 1–18, 10.1017/s1041610220001763. 32985398

[15] P. S. Sachdev , H. Brodaty , S. Reppermund , et al., “The Sydney Memory and Ageing Study (MAS): Methodology and Baseline Medical and Neuropsychiatric Characteristics of an Elderly Epidemiological Non‐Demented Cohort of Australians Aged 70–90 Years. Research Support, Non‐U.S. Gov't,” International Psychogeriatrics 22, no. 8 (December 2010): 1248–1264, 10.1017/S1041610210001067. 20637138

[16] CHeReL . Master Linkage Key Quality Assurance (Centre for Health Record Linkage, 2012).

[17] Australian Institute of Health and Welfare . “Data From: Australian Refined Diagnosis‐Related Groups (AR‐DRG) Data Cubes,” Cat. no. WEB 216, (n.d.), https://www.aihw.gov.au/reports/hospitals/ar‐drg‐data‐cubes2019.

[18] N. A. Kochan , M. J. Slavin , H. Brodaty , et al., “Effect of Different Impairment Criteria on Prevalence of ‘Objective’ Mild Cognitive Impairment in a Community Sample,” American Journal of Geriatric Psychiatry 18, no. 8 (2010): 711–722, 10.1097/JGP.0b013e3181d6b6a9. 21491632

[19] J. A. Yesavage , T. L. Brink , T. L. Rose , et al., “Development and Validation of a Geriatric Depression Screening Scale: A Preliminary Report,” Journal of Psychiatric Research 17, no. 1 (1982): 37–49, 10.1016/0022-3956(82)90033-4. 7183759

[20] B. Toson , L. A. Harvey , and J. C. Close , “The ICD‐10 Charlson Comorbidity Index Predicted Mortality But Not Resource Utilization Following Hip Fracture,” Journal of Clinical Epidemiology 68, no. 1 (January 2015): 44–51, 10.1016/j.jclinepi.2014.09.017. 25447352

[21] L. Falsetti , G. Viticchi , N. Tarquinio , et al., “Charlson Comorbidity Index as a Predictor of In‐Hospital Death in Acute Ischemic Stroke Among Very Old Patients: A Single‐Cohort Perspective Study,” Neurological Sciences 37, no. 9 (September 2016): 1443–1448, 10.1007/s10072-016-2602-1. 27166707

[22] K. A. S. Wickrama , T. K. Lee , C. W. O'Neal , and F. O. Lorenz , Higher‐Order Growth Curves and Mixture Modeling With Mplus: A Practical Guide (Routledge/Taylor & Francis Group, 2016): xix, 326‐xix, 326.

[23] H. Cham , E. Reshetnyak , B. Rosenfeld , and W. Breitbart , “Full Information Maximum Likelihood Estimation for Latent Variable Interactions With Incomplete Indicators,” Multivariate Behavioral Research 52, no. 1 (2017): 12–30, 10.1080/00273171.2016.1245600. 27834491 PMC5489914

[24] I. Hindmarch , H. Lehfeld , P. de Jongh , and H. Erzigkeit , “The Bayer Activities of Daily Living Scale (B‐ADL),” Dementia and Geriatric Cognit¡ve Disorders 9, no. Suppl 2 (1998): 20–26, 10.1159/000051195. 9718231

[25] ABS . Ageing in Australia: Census of Population and Housing 2001 (Australian Bureau of Statistics, 2003).

[26] J. N. Trollor , T. M. Anderson , P. S. Sachdev , H. Brodaty , and G. Andrews , “Prevalence of Mental Disorders in the Elderly: The Australian National Mental Health and Well‐Being Survey,” American Journal of Geriatric Psychiatry 15, no. 6 (June 2007): 455–466, 10.1097/JGP.0b013e3180590ba9. 17545446

[27] Admitted Patient Care 2016–17: Australian Hospital Statistics (AIHW, 2018).

[28] K. J. Anstey , S. M. Hofer , and M. A. Luszcz , “A Latent Growth Curve Analysis of Late‐Life Sensory and Cognitive Function Over 8 Years: Evidence for Specific and Common Factors Underlying Change,” Psychology and Aging 18, no. 4 (2003): 714–726, 10.1037/0882-7974.18.4.714. 14692859

[29] S. Reppermund , P. S. Sachdev , J. Crawford , et al., “The Relationship of Neuropsychological Function to Instrumental Activities of Daily Living in Mild Cognitive Impairment. Research Support, Non‐U.S. Gov't,” International Journal of Geriatric Psychiatry 26, no. 8 (August 2011): 843–852, 10.1002/gps.2612. 20845500

[30] P. A. Boyle , L. Yu , R. S. Wilson , K. Gamble , A. S. Buchman , and D. A. Bennett , “Poor Decision Making Is a Consequence of Cognitive Decline Among Older Persons Without Alzheimer's Disease or Mild Cognitive Impairment,” PLoS One 7, no. 8 (2012): e43647, 10.1371/journal.pone.0043647. 22916287 PMC3423371

[31] P. A. Boyle , L. Yu , R. S. Wilson , E. Segawa , A. S. Buchman , and D. A. Bennett , “Cognitive Decline Impairs Financial and Health Literacy Among Community‐Based Older Persons Without Dementia. Article,” Psychology and Aging 28, no. 3 (2013): 614–624, 10.1037/a0033103. 23957225 PMC3778113

[32] B. D. James , P. A. Boyle , L. Yu , S. D. Han , and D. A. Bennett , “Cognitive Decline Is Associated With Risk Aversion and Temporal Discounting in Older Adults Without Dementia,” PLoS One 10, no. 4 (2015): e0121900, 10.1371/journal.pone.0121900. 25838074 PMC4383618

[33] L. Yu , G. Mottola , R. S. Wilson , O. Valdes , D. A. Bennett , and P. A. Boyle , “Metamemory and Financial Decision Making in Older Adults Without Dementia. Article,” Neuropsychology 36, no. 1 (2022): 35–43, 10.1037/neu0000773. 34726461 PMC8758505

[34] D. M. Lipnicki , P. S. Sachdev , J. Crawford , et al., “Risk Factors for Late‐Life Cognitive Decline and Variation With Age and Sex in the Sydney Memory and Ageing Study,” PLoS One 8, no. 6 (2013): e65841, 10.1371/journal.pone.0065841. 23799051 PMC3683032

[35] B. Van Rompaey , M. M. Elseviers , M. J. Schuurmans , L. M. Shortridge‐Baggett , S. Truijen , and L. Bossaert , “Risk Factors for Delirium in Intensive Care Patients: A Prospective Cohort Study,” Critical Care 13, no. 3 (2009/05/20 2009): R77, 10.1186/cc7892. PMC2717440 19457226

[36] C. Dziegielewski , C. Skead , T. Canturk , et al., “Delirium and Associated Length of Stay and Costs in Critically Ill Patients,” Critical Care Research And Practice 2021 (2021): 6612187–6612188, 10.1155/2021/6612187. 33981458 PMC8088381

[37] S. K. Inouye , R. G. J. Westendorp , and J. S. Saczynski , “Delirium in Elderly People. Research Support, N.I.H., Extramural Research Support, Non‐U.S. Gov't Review,” Lancet. 383, no. 9920 (2014): 911–922, 10.1016/s0140-6736(13)60688-1. 23992774 PMC4120864

[38] R. Song , X. Fan , and J. Seo , “Physical and Cognitive Function to Explain the Quality of Life Among Older Adults With Cognitive Impairment: Exploring Cognitive Function as a Mediator,” BMC Psychology 11, no. 1 (February 2023): 51, 10.1186/s40359-023-01087-5. 36814329 PMC9948328

[39] M. E. Coleman , M. E. H. Roessler , S. Peng , et al., “Social Enrichment on the Job: Complex Work With People Improves Episodic Memory, Promotes Brain Reserve, and Reduces the Risk of Dementia,” Alzheimer's and Dementia: The Journal of the Alzheimer's Association 19, no. 6 (June 2023): 2655–2665, 10.1002/alz.13035. PMC10272079 37037592

[40] I. E. van de Vorst , I. Vaartjes , M. I. Geerlings , M. L. Bots , and H. L. Koek , “Prognosis of Patients With Dementia: Results From a Prospective Nationwide Registry Linkage Study in the Netherlands,” BMJ Open 5, no. 10 (October 2015): e008897, 10.1136/bmjopen-2015-008897. PMC4636675 26510729

[41] H. Kendig , C. Browning , R. Pedlow , Y. Wells , and S. Thomas , “Health, Social and Lifestyle Factors in Entry to Residential Aged Care: An Australian Longitudinal Analysis,” Age and Ageing 39, no. 3 (May 2010): 342–349, 10.1093/ageing/afq016. 20233734

[42] C. Cheng , H. Wan , P. Cong , et al., “Targeting Neuroinflammation as a Preventive and Therapeutic Approach for Perioperative Neurocognitive Disorders,” Journal of Neuroinflammation 19, no. 1 (December 2022): 297, 10.1186/s12974-022-02656-y. 36503642 PMC9743533

[43] Y. Arai , T. Suzuki , S. Jeong , and H. Ohta , “Prognosis of Home‐Cared or Hospital‐Treated Acute Fever in Older Adults: A Prospective Multicenter Case‐Control Study,” Geriatrics and Gerontology International 23, no. 5 (May 2023): 355–361, 10.1111/ggi.14577. 37012674

[44] B. Leff , L. Burton , S. L. Mader , et al., “Hospital at Home: Feasibility and Outcomes of a Program to Provide Hospital‐Level Care at Home for Acutely Ill Older Patients,” Annals of Internal Medicine 143, no. 11 (December 2005): 798–808, 10.7326/0003-4819-143-11-200512060-00008. 16330791

[45] G. A. Caplan , J. Coconis , and J. Woods , “Effect of Hospital in the Home Treatment on Physical and Cognitive Function: A Randomized Controlled Trial,” Journals of Gerontology ‐ Series A Biological Sciences and Medical Sciences 60, no. 8 (August 2005): 1035–1038, 10.1093/gerona/60.8.1035. 16127109

[46] M. R. Nadelson , R. D. Sanders , and M. S. Avidan , “Perioperative Cognitive Trajectory in Adults,” British Journal of Anaesthesia 112, no. 3 (2014): 440–451, 10.1093/bja/aet420. 24384981

[47] A. L. Gross , M. C. Power , M. S. Albert , et al., “Application of Latent Variable Methods to the Study of Cognitive Decline When Tests Change Over Time,” Epidemiology 26, no. 6 (November 2015): 878–887, 10.1097/EDE.0000000000000379. 26414855 PMC4819068

[48] E. L. Barr , A. M. Tonkin , T. A. Welborn , and J. E. Shaw , “Validity of Self‐Reported Cardiovascular Disease Events in Comparison to Medical Record Adjudication and a Statewide Hospital Morbidity Database: The AusDiab Study,” Internal Medicine Journal 39, no. 1 (January 2009): 49–53, 10.1111/j.1445-5994.2008.01864.x. 19290982

[49] G. Stoye and B. Zaranko , How Accurate Are Self‐Reported Diagnoses? Comparing Self‐Reported Health Events in the English Longitudinal Study of Ageing with Administrative Hospital Records. IFS Working Paper W20/13, 2020).

[50] P. Cheng , A. Gilchrist , K. M. Robinson , and L. Paul , “The Risk and Consequences of Clinical Miscoding Due to Inadequate Medical Documentation: A Case Study of the Impact on Health Services Funding,” Health Information Management Journal 38, no. 1 (2009): 35–46, 10.1177/183335830903800105. 19293434

