# Supplementary material for: All‐Cause Acute Illness Hospitalisations in the Preceding Two Years Are Associated With Cognitive Decline in Older Adults: The Sydney Memory and Ageing Study
Source: Int J Geriatr Psychiatry. 2025 May 1;40(5):e70077. doi: 10.1002/gps.70077 (PMC12045772; doi:10.1002/gps.70077)
Supplement: Supplementary file 1 — Supporting Information S1 [file GPS-40-e70077-s001.docx]

**Recent All-Cause Acute Illness Hospitalizations are associated with Cognitive Decline in Older Adults from the Sydney Memory and Ageing Study**

**Supplement**

**Method**

**Ethics**

MAS ethics approval was obtained from the University of New South Wales and South Eastern Sydney and Illawarra Area Health Service Ethics Committees (approval numbers 2015-20 HC14327 and 2020-25 HC190962). Access to linked New South Wales (NSW) Health data was approved by the NSW Population & Health Services Research Ethics Committee (AU RED Reference HREC/15/CIPHS/11 up to 2025) for 1, 026 of the 1, 037 (98.9%) individuals, on the basis that they had given consent for Medicare records to be accessed.

**Additional Linkage Information**

APDC linkage for the MAS sample had a false positive rate of 0.5%.The overall CHeReL false positive and false negative rates for APDC and Register of Births, Deaths and Marriages linkage are 0.3% and 0.5%, respectively ^1^.

**Table S1 MAS Participant Baseline Characteristics (n = 1,037) adapted from published MAS Methodology ^2^**

(**p* < .05 for significant difference between males and females on t-test for continuous variables or *χ*^2^ for categorical variables)

|  | | **Total**  **n = 1,037** | | **Males**  **n = 465 (45%)** | | **Females**  **n = 572 (55%)** | | **Differences** | |
| --- | --- | --- | --- | --- | --- | --- | --- | --- | --- |
| **Demographics** | | | | | | | | | |
| ***Continuous variables*** | | ***mean*** | ***SD*** | ***Mean*** | ***SD*** | ***mean*** | ***SD*** | ***t*** | ***p*** |
| Age | | 78.8 | 4.8 | 78.8 | 4.7 | 78.9 | 4.9 | -0.442 | .659 |
| Education | | 11.6 | 3.5 | 12.3 | 3.8 | 11.0 | 3.1 | 5.80 | .001* |
| ***Categorical variables*** | | ***n*** | ***% sample*** | ***n*** | ***% sample*** | ***n*** | ***% sample*** | *χ****^2^*** | ***p*** |
| Non-English Speaking Background | | 164 | 16 | 82 | 18 | 82 | 14 | 2.10 | .148 |
| English as primary language | | 976 | 94 | 435 | 94 | 541 | 95 | 0.493 | .482 |
| English as preferred language | | 972 | 94 | 432 | 93 | 540 | 94 | 0.985 | .321 |
| Region of Birth | Australia/New Zealand | 724 | 70 | 302 | 65 | 422 | 74 | 24.6 | <.001* |
|  | Europe/United Kingdom | 238 | 23 | 115 | 25 | 123 | 21 |  |  |
|  | Other | 75 | 7.2 | 48 | 10 | 27 | 4.7 |  |  |
| Occupation^[[1]](#footnote-1)^ | ABS Skill Level 1 | 451 | 43 | 283 | 61 | 168 | 29 | 169 | <.001* |
|  | ABS Skill Levels 2-5 | 586 | 57 | 182 | 39 | 404 | 71 |  |  |
| Living Arrangement | community alone | 486 | 47 | 141 | 30 | 345 | 60 | 99.2 | <.001* |
|  | community with others | 525 | 51 | 312 | 67 | 213 | 37 |  |  |
|  | other | 26 | 2.5 | 12 | 2.6 | 14 | 2.4 |  |  |
| **Physical health and Genetics** | | | | | | | | | |
| History of Diabetes Mellitus | | 126 of 1,035 | 12 | 78 of 462 | 17 | 48 of 573 | 8.4 | 17.3 | <.001* |
| Hypertension | | 629 of 1,033 | 61 | 268 of 460 | 58 | 361 of 573 | 63 | 2.41 | .121 |
| Hypolipidaemic drug | | 535 of 1,037 | 52 | 262 of 464 | 56 | 273 of 573 | 48 | 7.99 | .005* |
| Cholesterol ≥6.5 mmol/L | | 51 of 933 | 5.5 | 8 of 430 | 1.9 | 43 of 503 | 8.5 | 20.1 | <.001* |
| Previous Myocardial Infarct | | 118 of 1,022 | 12 | 84 of 460 | 18 | 34 of 562 | 6.0 | 36.9 | <.001* |
| Body Mass Index > 30 | | 202 of 1,010 | 22 | 102 of 453 | 23 | 120 of 557 | 22 | 0.138 | .710 |
| History of Cancer other than skin | | 140 of 1,035 | 14 | 66 of 464 | 14 | 74 of 571 | 13 | 0.350 | .554 |
| History of Thyroid disease | | 136 of 1,022 | 13 | 22 of 461 | 4.8 | 114 of 561 | 20 | 53.0 | <.001* |
| Obstructive Sleep Apnoea | | 57 of 1,023 | 5.6 | 40 of 459 | 8.7 | 17 of 564 | 3.0 | 15.6 | <.001* |
| Previous Cerebrovascular Accident | | 41 of 1,015 | 4.0 | 27 of 455 | 5.9 | 14 of 560 | 2.5 | 7.64 | .006* |
| Arthritis | | 555 of 1,014 | 55 | 229 of 456 | 50 | 326 of 558 | 58 | 6.82 | .009* |
| Regular Smoking previously | | 555 of 1,024 | 54 | 313 of 460 | 68 | 242 of 564 | 43 | 64.5 | <.001* |
| Alcohol ≥ 4 standard drinks/day | | 128 of 906 | 14 | 104 of 426 | 24 | 24 of 480 | 5.0 | 70.1 | <.001* |
| *APOE*4* homozygous | | 18 of 978 | 1.8 | 8 of 447 | 1.8 | 10 of 531 | 1.9 | 0.028 | .868 |
| **Mental Health** | | | | | | | | | |
| ≥ 6 on Geriatric Depression Scale | | 73 of 1,032 | 7.1 | 37 of 464 | 8.0 | 36 of 568 | 6.3 | 1.04 | .308 |
| ≥ 5 on Goldberg Anxiety Scale | | 91 of 1,007 | 9.0 | 32 of 451 | 7.1 | 59 of 556 | 11 | 3.75 | .053 |
| **Function** | | | | | | | | | |
| impaired on IADLs^[[2]](#footnote-2)^ | | 44 of 971 | 4.5 | 21 of 431 | 4.9 | 23 of 540 | 4.3 | 0.208 | .648 |
| **Cognition** | | | | | | | | | |
| ***Continuous variables*** | | ***mean*** | ***SD*** | ***Mean*** | ***SD*** | ***mean*** | ***SD*** | ***t*** | ***p*** |
| Mini-Mental State Examination | | 28.7 | 1.34 | 28.6 | 1.36 | 28.8 | 1.33 | -1.43 | .154 |
| IQ-CODE^[[3]](#footnote-3)^ (n = 938) | | 3.10 | 0.31 | 3.09 | 0.32 | 3.10 | 0.30 | -0.612 | .541 |
| ***Categorical variables*** | | ***n*** | ***% sample*** | ***n*** | ***% sample*** | ***n*** | ***% sample*** | ***X^2^*** | ***p*** |
| IQ-CODE > 3 | | 493 of 938 | 53 | 212 of 426 | 50 | 281of 512 | 55 | 2.44 | N/A |
| MCI^[[4]](#footnote-4)^ | Any type | 329 of 878 | 37 | 156 of 380 | 41 | 173 of 498 | 35 | 3.37 | .066 |

**Inclusion and Exclusion Criteria**

**Table S2 Sydney Memory and Ageing Study Description of Participants and Inclusion and Exclusion Criteria**

| **Inclusion** | - randomly recruited by letters sent to 8914 individuals on the federal electoral rolls for Kingsford-Smith and Wentworth - aged 70 to 90 years - have a Mini-Mental State Examination (MMSE) score ≥ 24 adjusted for age, education and non-English speaking background at study entry - have adequate spoken and written English to complete a psychometric assessment - be able to consent to participation - ideally required to have an informant who had contact with the participant for more than an hour per week and were able to answer questions regarding their cognition and function (94% had an appropriate informant) |
| --- | --- |
| **Exclusion** | - diagnosis of dementia: previous or after comprehensive MAS assessment - previous diagnosis of the following neurological or psychiatric conditions: schizophrenia, bipolar affective disorder, multiple sclerosis, motor neuron disease, developmental disability - progressive malignancy: active cancer or receiving treatment for cancer (not prostate cancer without metastasis and skin cancer) - psychotic symptoms - other medical or psychological conditions preventing completion of assessments |

**Table S3 MAS Neuropsychological test battery**

| **Domain** | **Test** | **Description** |
| --- | --- | --- |
| Premorbid Intelligence | National Adult Reading Test (NART) ^4^ | Not used in this project |
| Attention/ processing speed | Digit Symbol-Coding ^5^ | a test of coding according to a key to quickly fill in shapes paired with numbers |
|  | Trail Making Test A ^6^ | a task requiring numbers to be connected in numerical order as quickly as possible |
| Memory | Logical Memory Story A delayed recall ^7^ | a verbal memory task requiring the delayed recall of a narrative |
|  | Rey Auditory Verbal Learning Test ^8^   - total learning; trials 1-5 - short-term delayed recall; trial 6 - long-term delayed recall; trial 7 | verbal memory tasks requiring the recall of a list of 15 words |
|  | Benton Visual Retention Test recognition ^9^ | a visual memory task requiring the recognition of a geometric design |
| Language | Boston Naming Test – 30 items ^10^ | a confrontation naming test of visually presented objects and animals |
|  | Semantic Fluency (Animals) ^11^ | a speeded word generation task in a given semantic category |
| Visuo-spatial | Block Design ^12^ | a task requiring the copying of designs with blocks |
| Executive Function | Controlled Oral Word Association Test (Verbal Fluency Test using letters F-A-S) ^13^ | a speeded word generation task to a given initial letter and rule constraints |
|  | Trail Making Test B ^6^ | a task requiring numbers and letters to be connected in an alternating and sequential order as quickly as possible |
| Fine Motor | Grooved Pegboard Test ^14^ | not used in this project |

**Table S4 Exploratory Factor Analysis^[[5]](#footnote-5)^**

|  | **Factor** | | |
| --- | --- | --- | --- |
|  | **1** | **2** | **3** |
| Digit Symbol Substitution Test Total | .652 | -.005 | .136 |
| Trail Making test A time | -.771 | -.030 | .131 |
| Boston Naming Test total | .083 | .018 | .565 |
| Semantic Fluency (Animals) total | .034 | .042 | .641 |
| Trail Making Test B time | -.766 | -.031 | .002 |
| Controlled Oral Word Association Test | .052 | .014 | .496 |
| Rey Auditory Verbal Learning Test total number correct for trials 1-5 | .021 | .806 | .102 |
| Rey Auditory Verbal Learning Test: short-term delayed recall; trial 6 number correct | .022 | .949 | -0.56 |
| Rey Auditory Verbal Learning Test: long-term delayed recall; trial 7 number correct | .039 | .958 | -.108 |
| Logical Memory Story A delayed recall | -.041 | .408 | .270 |
| Benton Visual Retention Test recognition correct total | .362 | .058 | .153 |
| Block Design Total Score | .489 | -.069 | .204 |

**Table S5 Cognitive domain factor descriptives**

|  | whole sample | | | males | | | females | | | p^^[[6]](#footnote-6)^^ |
| --- | --- | --- | --- | --- | --- | --- | --- | --- | --- | --- |
| Language^^[[7]](#footnote-7)^^: Boston Naming Test, Semantic Fluency Animals, Controlled Oral Word Association Test | | | | | | | | | | |
|  | n | mean | SD | n | mean | SD | n | mean | SD |  |
| Wave 1 | 1,024 | 0.000 | 1.00 | 460 | 0.026 | 1.03 | 564 | -0.021 | 0.974 | .46 |
| Wave 2 | 877 | -0.109 | 0.99 | 404 | -0.123 | 1.01 | 473 | -0.098 | 0.974 | .71 |
| Wave 3 | 764 | -0.133 | 1.03 | 355 | -0.134 | 1.05 | 409 | -0.133 | 1.02 | .98 |
| Wave 4 | 653 | -0.182 | 1.09 | 296 | -0.193 | 1.10 | 357 | -0.173 | 1.09 | .87 |
| Memory: Logical Memory Story A delayed recall and Rey Auditory Verbal Learning Test | | | | | | | | | | |
| Wave 1 | 1,025 | 0.000 | 1.00 | 460 | -0.261 | 0.943 | 565 | 0.212 | 0.995 | <.001 |
| Wave 2 | 872 | -0.088 | 1.06 | 404 | -0.368 | 0.998 | 471 | 0.151 | 1.06 | <.001 |
| Wave 3 | 752 | -0.067 | 1.09 | 350 | -0.342 | 1.03 | 402 | 0.174 | 1.09 | <.001 |
| Wave 4 | 645 | -0.188 | 1.17 | 292 | -0.492 | 1.09 | 353 | 0.063 | 1.18 | <.001 |
| Executive/ Spatial Function: Trail Making Tests A and B, Digit Symbol-Coding, Benton Visual Retention Test, Block Design | | | | | | | | | | |
| Wave 1 | 1,023 | 0.000 | 1.00 | 460 | 0.056 | 1.01 | 563 | -0.046 | 0.996 | .11 |
| Wave 2 | 868 | -0.041 | 1.11 | 401 | -0.008 | 1.13 | 448 | -0.070 | 1.10 | .42 |
| Wave 3 | 749 | -0.153 | 1.12 | 351 | -0.146 | 1.11 | 403 | -0.159 | 1.13 | .87 |
| Wave 4 | 648 | -0.416 | 1.23 | 294 | -0.404 | 1.28 | 354 | -0.426 | 1.19 | .83 |

**Figure S1 Timing of cognitive assessments and hospitalizations for participant number 8 to provide an example of study predictors (hosp and cLOS) and outcomes (global cognition at each wave).**

**
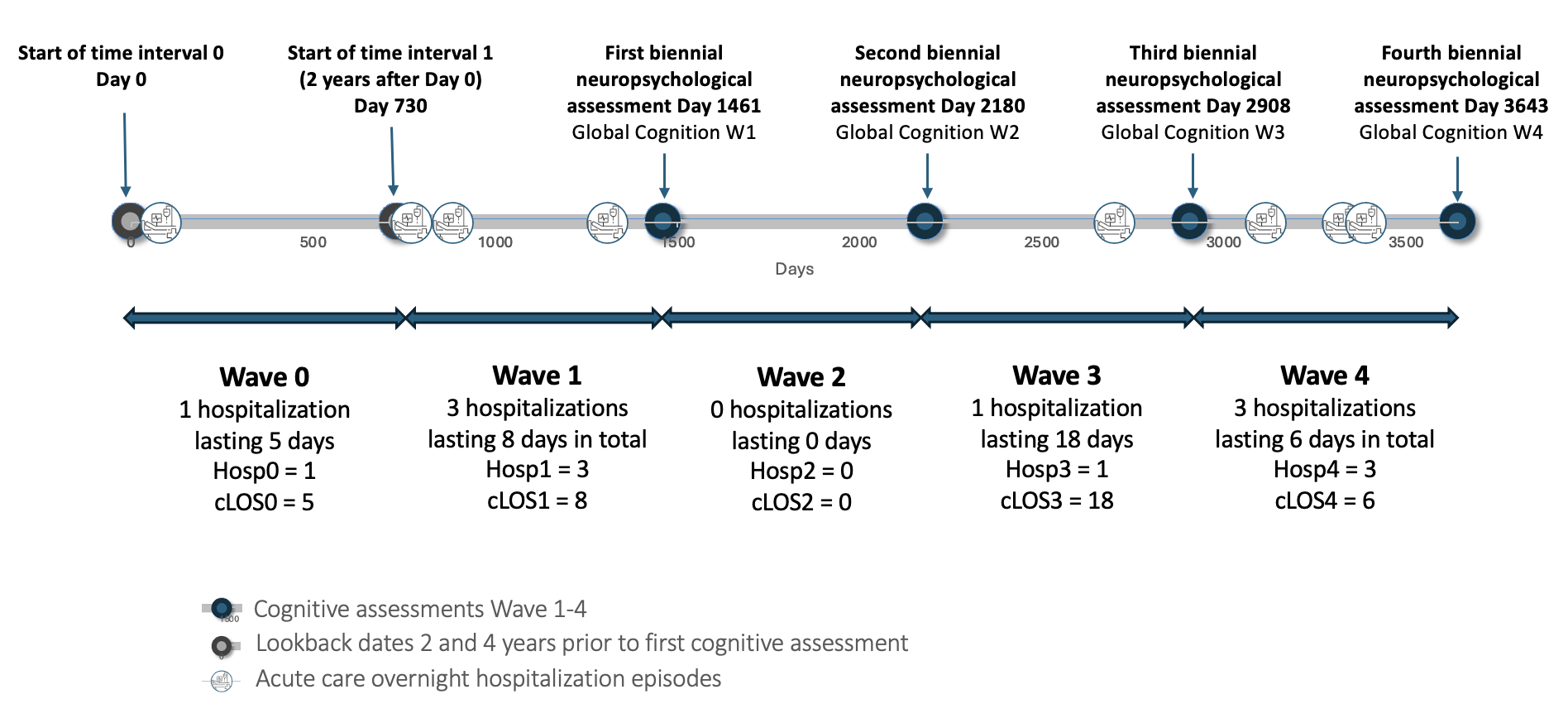
**

**Data Points for Figure S1**

**Figure S2 Longitudinal Confirmatory Factor Analysis for global cognition using cognition domain measures^15,16^** (W1 to W4: Cognitive Wave Assessments 1 to 4, Exec/Spat: Executive/ Spatial Cognition Factor)**^[[8]](#footnote-8)^**

**
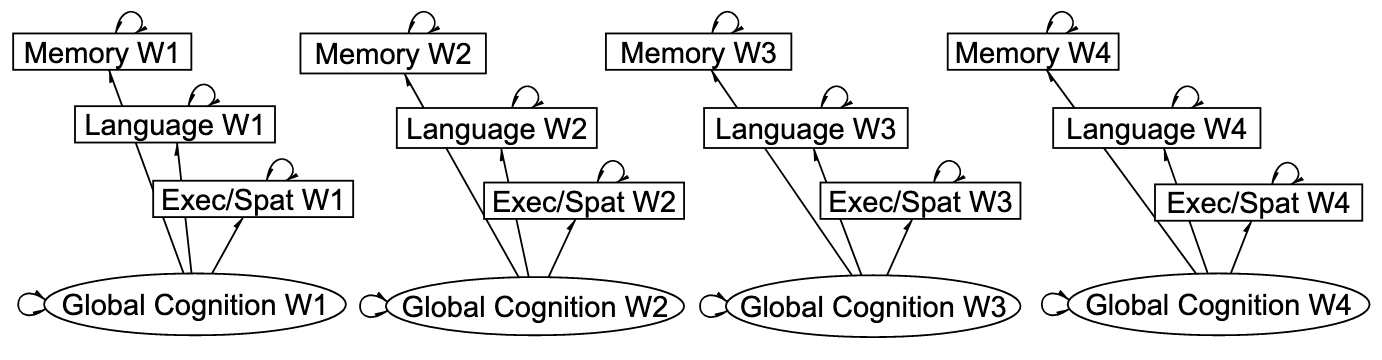
**

**Figure S3 Curve-of-Factors Model^1^ for global cognition using cognition domain measures^15,16^**

**
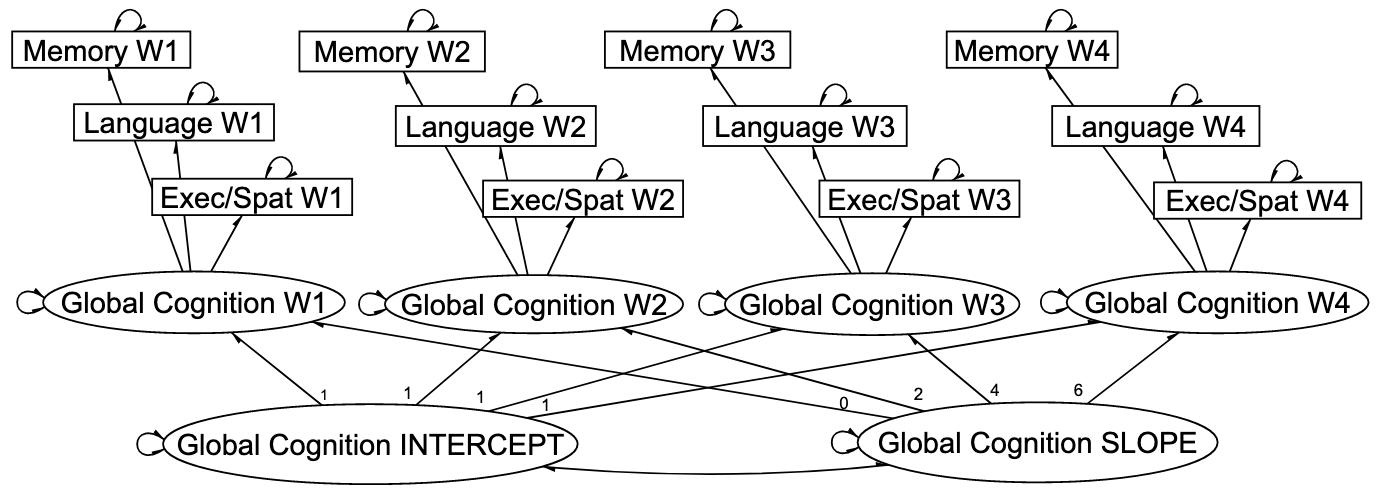
**

**Figure S4 Lagged Model using the LCFA (Figure S1) to estimate global cognition at each time point with five-interval hospitalization predictors (episodes: hosp0-4 and length of stay in days: los0-4) adjusted for age, sex and education.****^[[9]](#footnote-9)^** **^[[10]](#footnote-10)^**

**
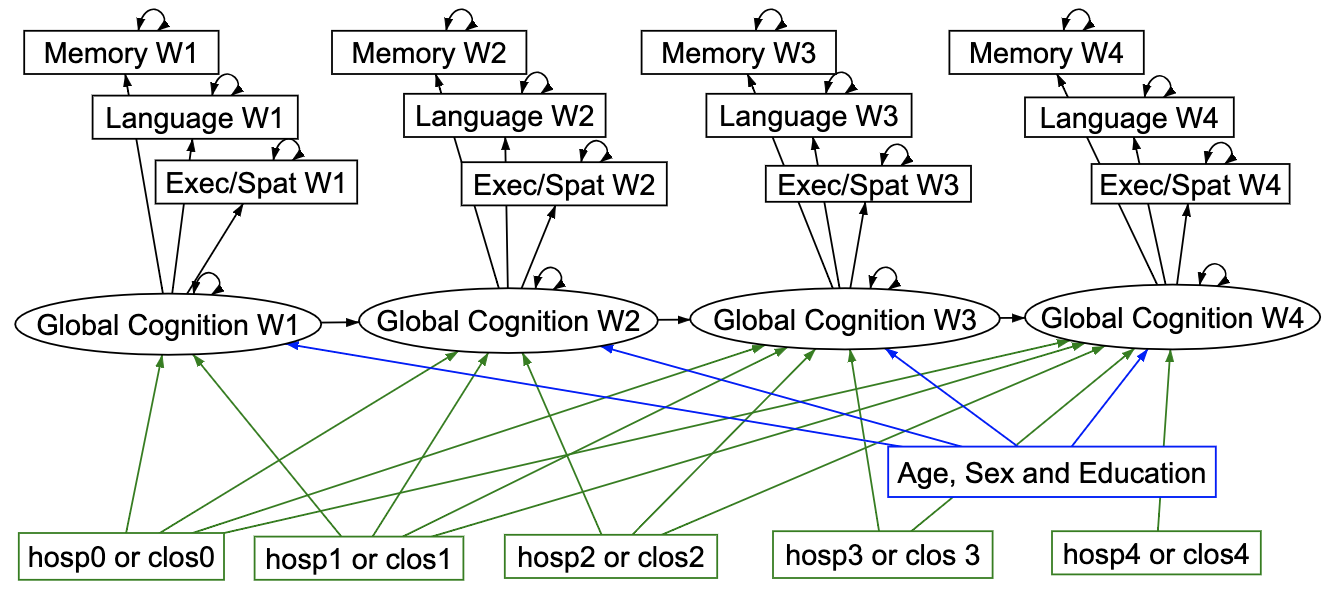
**

**Figure S5** Participation numbers for neuropsychological evaluation at each wave

**
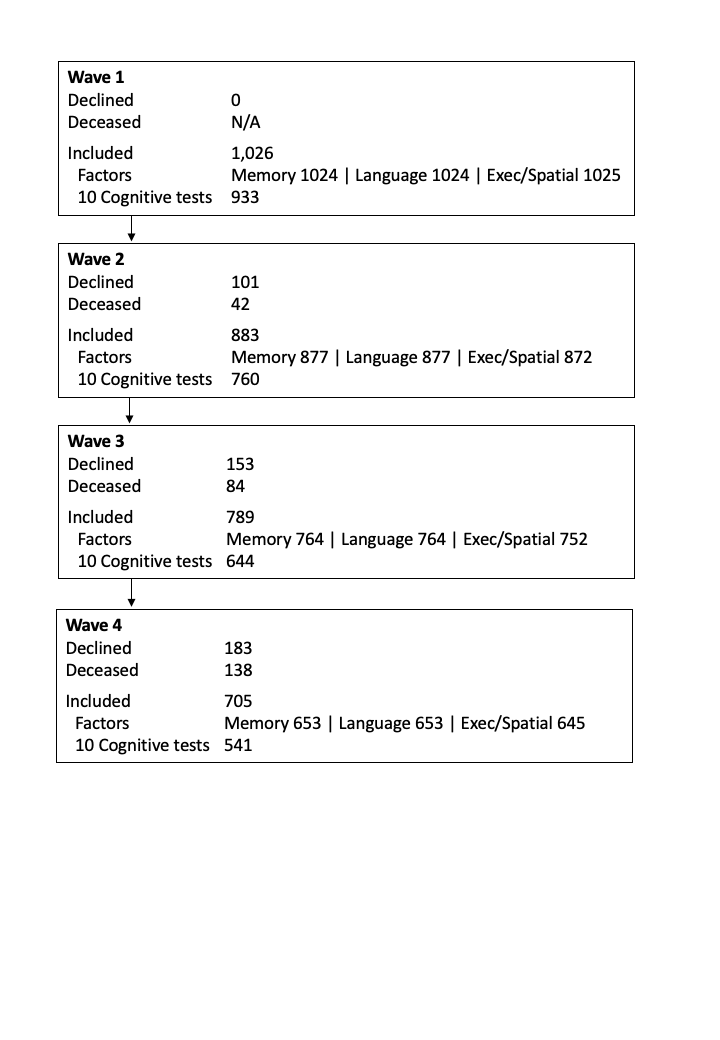
**

.

**Table S6 Measurement Invariance**

| **Model fit indices^[[11]](#footnote-11)^** | **χ^2^ (*df)*** | **CFI** | **ΔCFI^[[12]](#footnote-12)^** | **RMSEA (90% CI)** | **SRMR** |
| --- | --- | --- | --- | --- | --- |
| Configural LCFA model (unconstrained) | 35.3 (30) | 0.999 |  | 0.013 (0.000, 0.028) | 0.013 |
| Metric LCFA model (weak invariance) | 55.4 (36) | 0.998 | 0.001 | 0.023 (0.009, 0.034) | 0.019 |
| Scalar LCFA model (strong invariance) | 135 (42) | 0.989 | 0.009 | 0.046 (0.038, 0.055) | 0.034 |

**Measurement Invariance Results**

The LCFA models show that the observed variables demonstrated measurement invariance over time when used to estimate the global cognition latent factors at each time point with unconstrained factor loadings and weak, and strong invariance (all Δ Comparative Fit Index (CFI) < 0.01) (Table S6).

**Table S7 Comparison of Global Cognition Wave Scores and Intercept and Slope Estimates from Three Methods for Estimation/Calculation**^[[13]](#footnote-13)^

|  | Estimated from Three Cognition Factors | | Estimated from Five Cognitive Domains | | Calculated from Five Cognitive Domains | |
| --- | --- | --- | --- | --- | --- | --- |
| model fit indices | *CFI = 0.986, RMSEA = 0.050, SRMR = 0.033* | | *CFI =0.976, RMSEA =0.042, SRMR = 0.051* | | *CFI = 0.956, RMSEA = 0.090, SRMR = 0.078* | |
|  | **mean** | **SE** | **mean** | **SE** | **mean** | **SE** |
| latent intercept (*z-*score) | 0.00^[[14]](#footnote-14)^ | 1.00 | 0.00 | 1.00 | 0.00 | 1.00 |
| latent slope (*z-*score change/year) | -0.105 | 0.007 | -0.122 | 0.009 | -0.105 | 0.006 |

**Table S8 Hospitalization descriptives: Ten Years of Overnight Hospitalization Data (by hospitalization episode rather than by participant) compared to Australian Institute of Health and Welfare hospitalization data**

|  | **AIHW 2017/18**^[[15]](#footnote-15)^ | **Study Sample (n=1,026)** |
| --- | --- | --- |
|  | **n** | **n** |
| **TOTAL Hospitalizations** | 1,285,288 | 3,352^[[16]](#footnote-16)^ |
| Hospitalizations/person/year (mean ± SD) | 0.50 | 0.33 ± 0.34 |
| Emergency (%) |  | 1,479 (44.1) |
| Critical care (%) |  | 142 (4.2) |
| Central nervous system primary diagnosis (%) | 59,285 (4.6) | 124 (3.7) |
| Charlson Comorbidity Index^[[17]](#footnote-17)^ (mean ± SD) |  | 0.8 ± 1.3 |
| Length of stay per hospitalization (days) (mean ± SD) | 5.9 | 5.8 ± 7.7 |
| Days to next cognitive assessment^[[18]](#footnote-18)^ (mean ± SD) | Not applicable | 628 ± 518 |

**Table S9 Hospitalization descriptives in five approximately two-year time intervals separated by biennial cognition assessments and one lookback interval from two to four years preceding wave 1 assessment (interval 0)**

| Hospitalization episodes in five time intervals | | | | | | | | | | | | | |
| --- | --- | --- | --- | --- | --- | --- | --- | --- | --- | --- | --- | --- | --- |
|  | whole sample | | | | males | | | | females | | | | p^^[[19]](#footnote-19)^^ |
|  | n | mean | SD | range | n | mean | SD | range | n | mean | SD | range |  |
| interval 0^^[[20]](#footnote-20)^^ (hosp0^^[[21]](#footnote-21)^^) | 1,026 | 0.916 | 1.64 | 0-20 | 461 | 1.18 | 2.02 | 0-20 | 565 | 0.699 | 1.20 | 0-10 | <.001 |
| pre-Wave 1 (hosp1) | 1,026 | 0.550 | 1.00 | 0-10 | 461 | 0.625 | 1.06 | 0-7 | 565 | 0.488 | 0.950 | 0-10 | .03 |
| pre-Wave 2 (hosp2) | 883 | 0.647 | 1.15 | 0-10 | 405 | 0.738 | 1.26 | 0-10 | 478 | 0.569 | 1.04 | 0-7 | .03 |
| pre-Wave 3 (hosp3) | 789 | 0.791 | 1.36 | 0-14 | 367 | 0.929 | 1.57 | 0-14 | 422 | 0.671 | 1.13 | 0-8 | .01 |
| pre-Wave 4 (hosp4) | 705 | 0.926 | 1.49 | 0-17 | 316 | 1.01 | 1.47 | 0-9 | 389 | 0.856 | 1.50 | 0-17 | .16 |
| Cumulative length of stay (days) in five time intervals | | | | | | | | | | | | | |
| interval 0 (clos0) | 1,026 | 5.07 | 12.2 | 0-157 | 461 | 6.36 | 13.4 | 0-121 | 565 | 4.02 | 11.1 | 0-157 | .003 |
| pre-Wave 1 (clos1) | 1,026 | 2.95 | 8.33 | 0-116 | 461 | 3.24 | 8.77 | 0-116 | 565 | 2.71 | 7.96 | 0-94 | .32 |
| pre-Wave 2 (clos2) | 883 | 4.02 | 10.3 | 0-117 | 405 | 4.18 | 10.2 | 0-76 | 478 | 3.88 | 10.5 | 0-117 | .67 |
| pre-Wave 3 (clos3) | 789 | 4.60 | 11.1 | 0-105 | 367 | 5.04 | 11.4 | 0-91 | 422 | 4.21 | 10.8 | 0-105 | .30 |
| pre-Wave 4 (clos4) | 705 | 5.73 | 14.3 | 0-154 | 316 | 6.31 | 15.2 | 0-125 | 389 | 5.26 | 13.5 | 0-154 | .34 |

**Table S10 20 Most Common ICD-10-AM Diagnosis Codes for AIH in the MAS Sample from Wave 1 Assessment to 2011**


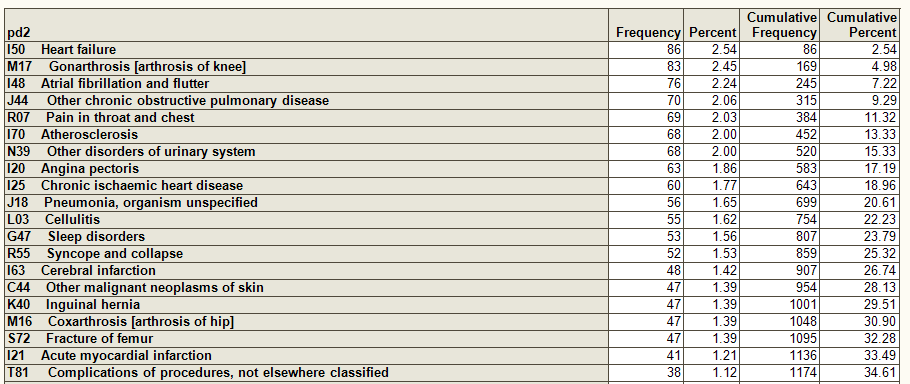


**Table S11 Central Nervous System Australian Refined-Diagnosis Related Groups (AR-DRG) Codes used for sensitivity analysis with CNS hospitalizations removed (Australian Institute of Health and Welfare (AIHW) figures compared to the Sydney Memory and Ageing Study sample (MAS))**

|  |  | AIHW 17/18 | MAS |
| --- | --- | --- | --- |
| **Surgical** | B01A Ventricular Shunt Revision, Major Complexity | 18 | 0 |
|  | B01B Ventricular Shunt Revision, Minor Complexity | 45 | 0 |
|  | B02A Cranial Procedures, Major Complexity | 799 | 2 |
|  | B02B Cranial Procedures, Intermediate Complexity | 1705 | 2 |
|  | B04A Extracranial Vascular Procedures, Major Complexity | 249 | 2 |
|  | B04B Extracranial Vascular Procedures, Intermediate Complexity | 449 | 13 |
|  | B67B (this patient would have also had a Surgical AR-DRG) | see Medical AR-DRG | 1 |
| TOTAL SURGICAL | | 3265 | 20 |
| **Other** | B42A Nervous System Disorders W Ventilator Support, Major Complexity | 165 | 0 |
|  | B42B Nervous System Disorders W Ventilator Support, Minor Complexity | 148 | 0 |
| **Medical** | B66A Nervous System Neoplasms, Major Complexity | 1396 | 0 |
|  | B66B Nervous System Neoplasms, Minor Complexity | 1033 | 1 |
|  | B67A Degenerative Nervous System Disorders, Major Complexity | 3472 | 0 |
|  | B67B Degenerative Nervous System Disorders, Intermediate Complexity | 1458 | 4 |
|  | B68A Multiple Sclerosis and Cerebellar Ataxia, Major Complexity | 135 | 0 |
|  | B68B Multiple Sclerosis and Cerebellar Ataxia, Minor Complexity | 28 | 1 |
|  | B69A TIA and Precerebral Occlusion, Major Complexity | 2690 | 12 |
|  | B69B TIA and Precerebral Occlusion, Minor Complexity | 4747 | 21 |
|  | B70A Stroke and Other Cerebrovascular Disorders, Major Complexity | 4120 | 4 |
|  | B70B Stroke and Other Cerebrovascular Disorders, Intermediate Complexity | 7340 | 18 |
|  | B70C Stroke and Other Cerebrovascular Disorders, Minor Complexity | 7857 | 21 |
|  | B70D Stroke and Other Cerebrovascular Disorders, Transferred <5 Days | 1757 | 1 |
|  | B72A Nervous System Infection Except Viral Meningitis, Major Complexity | 454 | 1 |
|  | B72B Nervous System Infection Except Viral Meningitis, Minor Complexity | 371 | 1 |
|  | B73Z Viral Meningitis | 33 | 0 |
|  | B74A Nontraumatic Stupor and Coma, Major Complexity | 455 | 2 |
|  | B74B Nontraumatic Stupor and Coma, Minor Complexity | 526 | 0 |
|  | B76A Seizures, Major Complexity | 2467 | 7 |
|  | B76B Seizures, Minor Complexity | 1691 | 2 |
|  | B78A Intracranial Injuries, Major Complexity | 2205 | 3 |
|  | B78B Intracranial Injuries, Minor Complexity | 2145 | 5 |
|  | B79A Skull Fractures, Major Complexity | 227 | 0 |
|  | B79B Skull Fractures, Minor Complexity | 110 | 0 |
|  | B80A Other Head Injuries, Major Complexity | 1859 | 0 |
|  | B80B Other Head Injuries, Minor Complexity | 1848 | 0 |
| TOTAL MEDICAL/OTHER | | 56020 | 104 |
| TOTAL CNS | | 59285 | 124 |

**Table S12 The Association of the Number of Hospitalizations in Five Time Intervals with Cognition Intercept and Slope, adjusted for Age, Sex and Education**

|  | **Adjusted for Age, Sex and Education** | | | | | | | |
| --- | --- | --- | --- | --- | --- | --- | --- | --- |
|  | *Model fit: CFI = 0.964, RMSEA = 0.047, SRMR = 0.068; R-squared for INTERCEPT = 0.337, for SLOPE = 0.112* | | | | | | | |
|  | **Estimated Effect on Cognition Intercept** | | | | **Estimated Effect on Cognition Slope** | | | |
|  | **b** | **SE** | ***p*** | **𝛃** | **b** | ***SE*** | ***p*** | **𝛃** |
| **Hospitalizations Interval 0 (hosp0)** | -0.019 | 0.026 | .459 | -0.025 | -0.002 | 0.005 | .780 | -0.018 |
| **Hospitalizations pre-Wave 1 (hosp1)** | 0.003 | 0.041 | .941 | 0.002 | 0.002 | 0.007 | .738 | 0.018 |
| **Hospitalizations pre-Wave 2 (hosp2)** |  |  |  |  | 0.001 | 0.008 | .873 | 0.011 |
| **Hospitalizations pre-Wave 3 (hosp3)** |  |  |  |  | -0.004 | 0.007 | .514 | -0.046 |
| **Hospitalizations pre-Wave 4 (hosp4)** |  |  |  |  | -0.010 | 0.004 | .019 | -0.115 |
| **Age** | -0.110 | 0.010 | <.001 | -0.430 | -0.009 | 0.001 | <.001 | -0.305 |
| **Female** | 0.252 | 0.090 | .005 | 0.102 | 0.000 | 0.013 | .973 | -0.002 |
| **Education** | 0.132 | 0.014 | <.001 | 0.372 | -0.002 | 0.002 | .296 | -0.052 |

**Table S13 The Association of Number of Days in Hospital in Five Time Intervals with Cognition Intercept and Slope, adjusted for Age, Sex and Education**

|  | **Adjusted for Age, Sex and Education** | | | | | | | |
| --- | --- | --- | --- | --- | --- | --- | --- | --- |
|  | *Model fit: CFI = 0.966, RMSEA = 0.046, SRMR = 0.064; R-squared for INTERCEPT = 0.339, for SLOPE = 0.138* | | | | | | | |
|  | **Estimated Effect on Cognition Intercept** | | | | **Estimated Effect on Cognition Slope** | | | |
|  | **b** | **SE** | ***p*** | **𝛃** | **b** | ***SE*** | ***p*** | **𝛃** |
| **Days in Hospital Interval 0 (los0)** | 0.001 | 0.004 | .739 | 0.012 | 0.000 | 0.001 | .555 | 0.041 |
| **Days in Hospital pre-Wave 1 (los1)** | -0.010 | 0.005 | .057 | -0.069 | 0.002 | 0.001 | .135 | 0.097 |
| **Days in Hospital pre-Wave 2 (los2)** |  |  |  |  | -0.001 | 0.001 | .219 | -0.090 |
| **Days in Hospital pre-Wave 3 (los3)** |  |  |  |  | -0.001 | 0.001 | .462 | -0.048 |
| **Days in Hospital pre-Wave 4 (los4)** |  |  |  |  | -0.002 | 0.000 | <.001 | -0.180 |
| **Age** | -0.110 | 0.009 | <.001 | -0.429 | -0.008 | 0.001 | <.001 | -0.294 |
| **Female** | 0.256 | 0.090 | .004 | 0.104 | 0.002 | 0.013 | .910 | 0.005 |
| **Education** | 0.131 | 0.014 | <.001 | 0.371 | -0.002 | 0.002 | .285 | -0.053 |

**Table S14 Additional covariate effects added separately one-by-one to basic covariate (Age/Sex/Education) CFM model hospitalization episode predictors in five time intervals (those with p<.10 selected for use in the final model). Full MPlus output for all models available on request.**

|  | **Estimated Effect on Latent Cognition Intercept** | | | **Estimated Effect on Latent Cognition Slope** | | |
| --- | --- | --- | --- | --- | --- | --- |
|  | **b** | **SE** | ***p*** | **b** | ***SE*** | ***p*** |
| **Non-English Speaking Background** | -0.995 | 0.126 | <.001 | 0.007 | 0.017 | .675 |
| **Vascular Condition** | 0.095 | 0.082 | .244 | 0.006 | 0.014 | .650 |
| **Other Cardiac** | -0.027 | 0.105 | .800 | -0.003 | 0.018 | .866 |
| **Kidney Disease** | -0.046 | 0.252 | .854 | 0.046 | 0.044 | .299 |
| **Asthma** | -0.046 | 0.159 | .771 | -0.008 | 0.028 | .765 |
| **Chronic Obstructive Pulmonary Disease** | 0.039 | 0.121 | .746 | -0.029 | 0.027 | .285 |
| **Anaemia** | -0.037 | 0.110 | .738 | -0.004 | 0.020 | .826 |
| **Thyroid Disease** | -0.027 | 0.110 | .808 | -0.010 | 0.021 | .634 |
| **Cancer** | 0.190 | 0.081 | .019 | 0.011 | 0.013 | .416 |
| **Cancer treated with Chemotherapy and /or Radiotherapy** | 0.227 | 0.143 | .112 | -0.010 | 0.024 | .675 |
| **Geriatric Depression Scale Score** | -0.082 | 0.020 | <.001 | 0.002 | 0.003 | .571 |
| **Apolipoprotein E ε4 allele** | -0.200 | 0.097 | .040 | -0.046 | 0.016 | .005 |
| **Years of Diabetes Mellitus** | -0.013 | 0.005 | .010 | -0.001 | 0.001 | .578 |
| **Smoking Pack years** | 0.003 | 0.002 | .038 | 0.000 | 0.000 | .164 |
| **Alcohol > 6 standard drinks/week** | 0.141 | 0.049 | .004 | 0.005 | 0.008 | .532 |
| **Self-rated General Health** | 0.173 | 0.044 | <.001 | 0.004 | 0.007 | .558 |
| **Body Mass Index** | 0.006 | 0.009 | .508 | 0.000 | 0.001 | .985 |
| **Obstructive Sleep Apnoea** | 0.181 | 0.182 | .318 | 0.012 | 0.024 | .598 |
| **Central Nervous System Disease** | 0.029 | 0.081 | .717 | 0.000 | 0.014 | .989 |

**Table S15 Additional covariate effects added separately one-by-one to basic covariate (Age/Sex/Education) CFM model with number of days in hospital predictors in five time intervals (those with p<.10 selected for use in fully adjusted model). Full MPlus output for all models available on request.**

|  | **Estimated Effect on Latent Cognition Intercept** | | | **Estimated Effect on Latent Cognition Slope** | | |
| --- | --- | --- | --- | --- | --- | --- |
|  | **b** | **SE** | ***p*** | **b** | ***SE*** | ***p*** |
| **Non-English Speaking Background** | -0.991 | 0.125 | <.001 | 0.004 | 0.017 | .802 |
| **Vascular Condition** | 0.100 | 0.082 | .218 | 0.006 | 0.014 | .678 |
| **Other Cardiac** | -0.007 | 0.104 | .943 | -0.001 | 0.018 | .976 |
| **Kidney Disease** | -0.031 | 0.251 | .901 | 0.045 | 0.038 | .246 |
| **Asthma** | -0.042 | 0.160 | .793 | -0.003 | 0.028 | .917 |
| **Chronic Obstructive Pulmonary Disease** | 0.050 | 0.120 | .677 | -0.024 | 0.028 | .391 |
| **Anaemia** | -0.020 | 0.089 | .826 | -0.025 | 0.149 | .867 |
| **Thyroid Disease** | -0.022 | 0.110 | .843 | -0.010 | 0.021 | .629 |
| **Cancer** | 0.192 | .081 | .018 | 0.010 | 0.013 | .459 |
| **Cancer treated with Chemotherapy and /or Radiotherapy** | 0.243 | 0.142 | .088 | -0.013 | 0.024 | .602 |
| **Geriatric Depression Scale Score** | -0.077 | 0.020 | <.001 | 0.002 | 0.003 | .617 |
| **Apolipoprotein E ε4 allele** | -0.203 | 0.097 | .037 | -0.049 | 0.016 | .006 |
| **Years of Diabetes Mellitus** | -0.012 | 0.005 | .010 | -0.001 | 0.001 | .636 |
| **Smoking Pack years** | 0.003 | 0.002 | .033 | 0.000 | 0.000 | .265 |
| **Alcohol > 6 standard drinks/week** | 0.134 | 0.049 | .006 | 0.007 | 0.008 | .398 |
| **Self-rated General Health** | 0.163 | 0.044 | <.001 | 0.005 | 0.007 | .498 |
| **Body Mass Index** | 0.006 | 0.009 | .500 | 0.000 | 0.001 | .930 |
| **Obstructive Sleep Apnoea** | 0.182 | 0.183 | .319 | 0.006 | 0.024 | .786 |
| **Central Nervous System Disease** | 0.029 | 0.081 | .723 | 0.001 | 0.014 | .961 |

**Sensitivity analyses removing CNS hospitalizations**

**Table S16 Sensitivity Analysis removing Hospitalization episodes with Primary CNS Diagnoses**

(fully adjusted five-time interval model showing hospitalization predictor effects only)

|  | **Adjusted for Age, Sex, Education and Covariates with p < .10** | | | | | | | |
| --- | --- | --- | --- | --- | --- | --- | --- | --- |
|  | *Model fit: CFI = 0.954, RMSEA = 0.041, SRMR = 0.064; R-squared INTERCEPT = 0.450, SLOPE = 0.145* | | | | | | | |
|  | **Estimated Effect on Cognition Intercept** | | | | **Estimated Effect on Cognition Slope** | | | |
|  | **b** | **SE** | ***p*** | **𝛃** | **b** | ***SE*** | ***p*** | **𝛃** |
| **Hospitalizations Interval 0 (hosp0)** | -0.034 | 0.025 | .179 | -0.041 | -0.003 | 0.006 | .667 | -0.028 |
| **Hospitalizations pre-Wave 1 (hosp1)** | 0.051 | 0.044 | .243 | 0.037 | 0.005 | 0.008 | .553 | 0.032 |
| **Hospitalizations pre-Wave 2 (hosp2)** |  |  |  |  | 0.002 | 0.009 | .794 | -0.018 |
| **Hospitalizations pre-Wave 3 (hosp3)** |  |  |  |  | -0.002 | 0.008 | .763 | -0.022 |
| **Hospitalizations pre-Wave 4 (hosp4)** |  |  |  |  | -0.013 | 0.005 | .007 | -0.134 |

**Table S17 Sensitivity Analysis removing Number of Days in Hospital with Primary CNS Diagnoses**

(fully adjusted five-time interval model showing hospitalization predictor effects only)

|  | **Adjusted for Age, Sex, Education and Covariates with p < .10** | | | | | | | |
| --- | --- | --- | --- | --- | --- | --- | --- | --- |
|  | *Model fit: CFI = 0.955, RMSEA = 0.040, SRMR = 0.060; R-squared for INTERCEPT = 0.447, SLOPE = 0.170* | | | | | | | |
|  | **Estimated Effect on Cognition Intercept** | | | | **Estimated Effect on Cognition Slope** | | | |
|  | **b** | **SE** | ***p*** | **𝛃** | **b** | ***SE*** | ***p*** | **𝛃** |
| **Days in Hospital Interval 0 (los0)** | -0.001 | 0.004 | .685 | -0.013 | 0.001 | 0.001 | .559 | 0.041 |
| **Days in Hospital pre-Wave 1 (los1)** | -0.004 | 0.005 | .496 | -0.022 | 0.002 | 0.001 | .103 | 0.109 |
| **Days in Hospital pre-Wave 2 (los2)** |  |  |  |  | -0.001 | 0.001 | .363 | -0.067 |
| **Days in Hospital pre-Wave 3 (los3)** |  |  |  |  | -0.001 | 0.001 | .520 | -0.043 |
| **Days in Hospital pre-Wave 4 (los4)** |  |  |  |  | -0.002 | 0.001 | <.001 | -0.190 |

**Lagged Models to look for Recency Effects at Each Time Point (Figures S5-S8)**

**Table S18 Lagged Model showing the association of Number of Hospitalizations in Five Time Intervals with Global Cognition at each time point, adjusted for age, sex and education.^[[22]](#footnote-22)^**

| **Estimated Effects** | **Adjusted for Age, Sex, Education** | | | | | | | | | | | |
| --- | --- | --- | --- | --- | --- | --- | --- | --- | --- | --- | --- | --- |
|  | *Model fit: CFI = 0.976, RMSEA = 0.044, SRMR = 0.042; R-squared GCOG1 = 0.343, GCOG2 = 0.937, GCOG3 = 0.919, GCOG4 = 0.946* | | | | | | | | | | | |
|  | **Global Cognition Wave 1**  **(GCOG1)** | | | **Global Cognition Wave 2**  **(GCOG2)****^[[23]](#footnote-23)^** | | | **Global Cognition Wave 3**  **(GCOG3)^2^** | | | **Global Cognition Wave 4**  **(GCOG4)^2^** | | |
|  | **b** | **SE** | ***p*** | **b** | ***SE*** | ***p*** | **b** | ***SE*** | ***p*** | **b** | ***SE*** | ***p*** |
| **Hospitalizations Interval 0 (hosp0)** | -0.020 | 0.026 | .444 | 0.009 | 0.022 | .684 | -0.008 | 0.020 | .693 | -0.018 | 0.029 | .538 |
| **Hospitalizations pre-Wave 1 (hosp1)** | 0.004 | 0.042 | .933 | 0.005 | 0.032 | .864 | -0.018 | 0.032 | .582 | 0.026 | 0.035 | .450 |
| **Hospitalizations pre-Wave 2 (hosp2)** |  |  |  | -0.061 | 0.027 | .024 | 0.083 | 0.037 | .026 | -0.002 | 0.034 | .942 |
| **Hospitalizations pre-Wave 3 (hosp3)** |  |  |  |  |  |  | -0.042 | 0.023 | .066 | 0.020 | 0.028 | .478 |
| **Hospitalizations pre-Wave 4 (hosp4)** |  |  |  |  |  |  |  |  |  | -0.041 | 0.021 | .051 |
| **Age** | -0.110 | 0.010 | <.001 | -0.011 | 0.007 | .115 | -0.007 | 0.008 | .393 | -0.010 | 0.008 | .234 |
| **Female Sex** | 0.247 | 0.092 | .007 | -0.007 | 0.051 | .890 | 0.000 | 0.058 | .994 | -0.065 | 0.060 | .277 |
| **Education** | 0.132 | 0.014 | <.001 | -0.015 | 0.009 | .096 | -0.013 | 0.010 | .204 | -0.011 | 0.010 | .302 |

**Table S19 Lagged Model showing the association of Number of Days in Hospital in Five Time Intervals with Global Cognition at each time point, adjusted for age, sex and education.**

| **Estimated Effects** | **Adjusted for Age, Sex, Education** | | | | | | | | | | | |
| --- | --- | --- | --- | --- | --- | --- | --- | --- | --- | --- | --- | --- |
|  | *Model fit: CFI = 0.975, RMSEA = 0.045, SRMR = 0.042.*  *R-squared GCOG1 = 0.346, GCOG2 = 0.941, GCOG3 = 0.918, GCOG4 = 0.948* | | | | | | | | | | | |
|  | **Global Cognition Wave 1**  **(GCOG1)** | | | **Global Cognition Wave 2**  **(GCOG2)^[[24]](#footnote-24)^** | | | **Global Cognition Wave 3**  **(GCOG3)^2^** | | | **Global Cognition Wave 4**  **(GCOG4)^2^** | | |
|  | **b** | **SE** | ***p*** | **b** | ***SE*** | ***p*** | **b** | ***SE*** | ***p*** | **b** | ***SE*** | ***p*** |
| **Days in Hospital Interval 0 (los0)** | 0.002 | 0.004 | .680 | 0.001 | 0.004 | .848 | -0.001 | 0.003 | .751 | 0.004 | 0.004 | .330 |
| **Days in Hospital pre-Wave 1 (los1)** | -0.010 | 0.005 | .053 | 0.006 | 0.004 | .176 | 0.000 | 0.004 | .915 | 0.005 | 0.004 | .283 |
| **Days in Hospital pre-Wave 2 (los2)** |  |  |  | -0.012 | 0.004 | .003 | 0.009 | 0.004 | .032 | 0.000 | 0.004 | .934 |
| **Days in Hospital pre-Wave 3 (los3)** |  |  |  |  |  |  | -0.005 | 0.004 | .215 | 0.002 | 0.003 | .608 |
| **Days in Hospital pre-Wave 4 (los4)** |  |  |  |  |  |  |  |  |  | -0.007 | 0.002 | <.001 |
| **Age** | -0.110 | 0.010 | <.001 | -0.009 | 0.007 | .173 | -0.007 | 0.008 | .368 | -0.010 | 0.008 | .222 |
| **Female Sex** | 0.251 | 0.091 | .006 | -0.001 | 0.052 | .979 | 0.002 | 0.058 | .969 | -0.062 | 0.060 | .304 |
| **Education** | -0.010 | 0.005 | .053 | -0.016 | 0.009 | .082 | -0.013 | 0.010 | .198 | -0.010 | 0.010 | .303 |

**Interactions with Age, Sex, Education and Apolipoprotein E ε4 allele (*APOE*4)***

**Table S20 Significant^[[25]](#footnote-25)^ Interaction Effects between Hospitalization Episodes and Age, Education, Sex and *APOE*4***

|  | **Adjusted for Age, Sex and Education** | | | | | | | | | |
| --- | --- | --- | --- | --- | --- | --- | --- | --- | --- | --- |
|  | **(Cognition Intercept)** | | | | **Estimated Effect on Cognition Slope** | | | | | |
|  | **b** | **SE** | ***p*** | ***𝛃*** | **b** | ***SE*** | ***p*** | ***𝛃*** | **lower covariate value (-1 SD)^[[26]](#footnote-26)^**  **(*p*-value)** | **higher covariate value (+1 SD)**  **(*p*-value)** |
| **Hospitalization pre-Wave 2 and Age (H2_AGE)** |  |  |  |  | 0.004 | 0.002 | .017 | 0.177 | -0.090 (*.019*) | 0.091 (.*021*) |

**Table S21 Significant Interaction Effects between Number of Days in Hospital and Age, Education, Sex and *APOE*4***

|  | **Adjusted for Age, Sex and Education** | | | | | | | | | | | |
| --- | --- | --- | --- | --- | --- | --- | --- | --- | --- | --- | --- | --- |
|  | **Estimated Effect on Cognition Intercept** | | | | | | **Estimated Effect on Cognition Slope** | | | | | |
|  | **b** | **SE** | ***p*** | ***𝛃*** | **lower covariate value**  **(*p*-value)** | **higher covariate value**  **(*p*-value)** | **b** | ***SE*** | ***p*** | ***𝛃*** | **lower covariate value**  **(*p*-value)** | **higher covariate value**  **(*p*-value)** |
| None were significant | | | | | | | | | | | | |

**Models with Charlson Comorbidity Index^[[27]](#footnote-27)^ (CCI)**

**Table S22 The Association of the Number of Hospitalizations in Five Time Intervals with Cognition Intercept and Slope, adjusted for Age, Sex, Education and CCI**

|  | **Adjusted for Age, Sex and Education****^[[28]](#footnote-28)^** | | | | | | | |
| --- | --- | --- | --- | --- | --- | --- | --- | --- |
|  | *Model fit: CFI = 0.963, RMSEA = 0.045, SRMR = 0.068; R-squared for INTERCEPT = 0.337, for SLOPE = 0.115* | | | | | | | |
|  | **Estimated Effect on Cognition Intercept** | | | | **Estimated Effect on Cognition Slope** | | | |
|  | **b** | **SE** | ***p*** | **𝛃** | **b** | ***SE*** | ***p*** | **𝛃** |
| **Hospitalizations Interval 0 (hosp0)** | -0.018 | 0.025 | .472 | -0.024 | -0.002 | 0.005 | .707 | -0.025 |
| **Hospitalizations pre-Wave 1 (hosp1)** | 0.015 | 0.047 | .485 | 0.743 | -0.001 | 0.009 | .899 | -0.008 |
| **Hospitalizations pre-Wave 2 (hosp2)** |  |  |  |  | 0.001 | 0.008 | .898 | 0.009 |
| **Hospitalizations pre-Wave 3 (hosp3)** |  |  |  |  | -0.003 | 0.007 | .693 | -0.039 |
| **Hospitalizations pre-Wave 4 (hosp4)** |  |  |  |  | -0.006 | 0.005 | .206 | -0.073 |
| **CCI in pre-Wave 1 interval** | -0.021 | 0.045 | .643 | -0.019 | 0.006 | 0.009 | .475 | 0.049 |
| **CCI in pre-Wave 4 interval** |  |  |  |  | -0.008 | 0.005 | .103 | -0.090 |

**Table S23 The Association of Number of Days in Hospital in Five Time Intervals with Cognition Intercept and Slope, adjusted for Age, Sex, Education and CCI**

|  | **Adjusted for Age, Sex and Education** | | | | | | | |
| --- | --- | --- | --- | --- | --- | --- | --- | --- |
|  | *Model fit: CFI = 0.964, RMSEA = 0.044, SRMR = 0.064; R-squared for INTERCEPT = 0.340, for SLOPE = 0.138* | | | | | | | |
|  | **Estimated Effect on Cognition Intercept** | | | | **Estimated Effect on Cognition Slope** | | | |
|  | **b** | **SE** | ***p*** | **𝛃** | **b** | ***SE*** | ***p*** | **𝛃** |
| **Days in Hospital Interval 0 (los0)** | 0.001 | 0.004 | .754 | -0.011 | 0.000 | 0.001 | .592 | 0.038 |
| **Days in Hospital pre-Wave 1 (los1)** | -0.012 | 0.006 | .046 | -0.079 | 0.002 | 0.001 | .179 | 0.093 |
| **Days in Hospital pre-Wave 2 (los2)** |  |  |  |  | -0.001 | 0.001 | .224 | -0.090 |
| **Days in Hospital pre-Wave 3 (los3)** |  |  |  |  | -0.001 | 0.001 | .509 | -0.043 |
| **Days in Hospital pre-Wave 4 (los4)** |  |  |  |  | -0.001 | 0.001 | .007 | -0.148 |
| **CCI in pre-Wave 1 interval** | 0.023 | 0.042 | .579 | 0.021 | 0.001 | 0.007 | .863 | 0.010 |
| **CCI in pre-Wave 4 interval** |  |  |  |  | -0.006 | 0.005 | .201 | -0.063 |

**Table S24 Lagged Model with Fixed paths: showing the Association of Number of Hospitalizations in Five Time Intervals with the most recent interval and earlier intervals fixed to be equal, on global cognition at each time point, adjusted for age, sex, education****^[[29]](#footnote-29)^ and CCI and the difference between recent and non-recent paths.**

| **Estimated Effects** | **Adjusted for Age, Sex, Education** | | |
| --- | --- | --- | --- |
|  | **Global Cognition at each timepoint** | | |
|  | **b** | **SE** | ***p*** |
| **Fixed path for hospitalizations in earlier time interval(s)** | 0.005 | 0.006 | .391 |
| **Fixed path for hospitalizations in the most recent time interval** | -0.025 | 0.015 | .096 |
| *Model fit: CFI = 0.976, RMSEA = 0.036, SRMR = 0.039; R-squared GCOG1 = 0.342, GCOG2 = 0.937, GCOG3 = 0.916, GCOG4 = 0.946* | | | |
| **Wald test for difference between recent and earlier paths χ^2^  = 2.941 *df* = 1 *p*-value = .086** | | | |

**Table S25 Lagged Model with Fixed paths: showing the association of Number of Days in Hospital in Five Time Intervals with the most recent interval and earlier intervals fixed to be equal, on global cognition at each time point, adjusted for age, sex, education1 and CCI and the difference between recent and non-recent paths.**

| **Estimated Effects** | **Adjusted for Age, Sex, Education** | | |
| --- | --- | --- | --- |
|  | **Global Cognition at each timepoint** | | |
|  | **b** | **SE** | ***p*** |
| **Fixed path for hospitalizations in earlier time interval(s)** | 0.002 | 0.001 | .005 |
| **Fixed path for hospitalizations in the most recent time interval** | -0.007 | 0.002 | <.001 |
| *Model fit: CFI = 0.975, RMSEA = 0.037, SRMR = 0.040; R-squared GCOG1 = 0.344, GCOG2 = 0.939, GCOG3 = 0.916, GCOG4 = 0.948* | | | |
| **Wald test for difference between recent and earlier paths** χ^2^  = 16.058 *df* = 1 *p*-value <.001 | | | |

**Table S26 The Association of Cognition Intercept and Slope with CCI adjusted for age, sex, education *without hospitalization variables*.**

|  | **Adjusted for Age, Sex and Education** | | | | | | | |
| --- | --- | --- | --- | --- | --- | --- | --- | --- |
|  | *Model fit: CFI = 0.970, RMSEA = 0.051, SRMR = 0.057; R-squared for INTERCEPT = 0.339, for SLOPE = 0.113* | | | | | | | |
|  | **Estimated Effect on Cognition Intercept** | | | | **Estimated Effect on Cognition Slope** | | | |
|  | **b** | **SE** | ***p*** | **𝛃** | **b** | ***SE*** | ***p*** | **𝛃** |
| **CCI in pre-Wave 1 interval** | -0.020 | 0.039 | .611 | -0.017 | 0.004 | 0.007 | .594 | 0.030 |
| **CCI in pre-Wave 4 interval** |  |  |  |  | -0.013 | 0.004 | .003 | -0.138 |

**Table S27 Lagged Model with the effect of maximum CCI on global cognition at each time point, adjusted for age, sex, education^[[30]](#footnote-30)^ *without hospitalization predictors*.**

| **Estimated Effects** | **Adjusted for Age, Sex and Education** | | |
| --- | --- | --- | --- |
|  | **Global Cognition at each timepoint** | | |
|  | **b** | **SE** | ***p*** |
| **CCI in pre-Wave 1 interval** | -0.014 | 0.038 | .719 |
| **CCI in pre-Wave 2 interval** | -0.051 | 0.026 | .046 |
| **CCI in pre-Wave 3 interval** | -0.033 | 0.021 | .110 |
| **CCI in pre-Wave 4 interval** | -0.019 | 0.019 | .298 |
| *Model fit: CFI = 0.975, RMSEA = 0.045, SRMR = 0.045; R-squared GCOG1 = 0.342, GCOG2 = 0.937, GCOG3 = 0.916, GCOG4 = 0.945* | | | |
| **Wald test for difference between recent and earlier paths χ^2^  =** 20.355 *df* = 1 *p*-value <.001 | | | |

**Table S28 Significant Interaction Effects between Hospitalization Episodes in the pre-Wave 4 interval and Charlson Comorbidity Index with one-year lookback (maximum in pre-Wave 4 interval) adjusting for Age, Education and Sex**

|  | **Adjusted for Age, Sex and Education** | | | | | | | | | |
| --- | --- | --- | --- | --- | --- | --- | --- | --- | --- | --- |
|  | **(Cognition Intercept)** | | | | **Estimated Effect on Cognition Slope** | | | | | |
|  | **b** | **SE** | ***p*** | ***𝛃*** | **b** | ***SE*** | ***p*** | ***𝛃*** | **lower covariate value (-1 SD)^[[31]](#footnote-31)^**  **(*p*-value)** | **higher covariate value (+1 SD)**  **(*p*-value)** |
| **CCI in pre-Wave 4 interval** |  |  |  |  | 0.004 | 0.002 | .035 | .120 | -0.009  (*.126*) | 0.001  (*.875*) |

**Table S29 Significant Interaction Effects between Number of Days in Hospital in the pre-Wave 4 interval and Charlson Comorbidity Index with one-year lookback (maximum in pre-Wave 4 interval) adjusting for Age, Education and Sex**

|  | **Adjusted for Age, Sex and Education** | | | | | | | | | |
| --- | --- | --- | --- | --- | --- | --- | --- | --- | --- | --- |
|  | **(Cognition Intercept)** | | | | **Estimated Effect on Cognition Slope** | | | | | |
|  | **b** | **SE** | ***p*** | ***𝛃*** | **b** | ***SE*** | ***p*** | ***𝛃*** | **lower covariate value (-1 SD)^[[32]](#footnote-32)^**  **(*p*-value)** | **higher covariate value (+1 SD)**  **(*p*-value)** |
| **CCI in pre-Wave 4 interval** |  |  |  |  | 0.000 | 0.000 | .471 | 0.004 | non-significant | |

**Figure S6 Maximum CCI in each time interval (CCI1YLB0-CCI1YLB4) Correlations with Hospitalization Episode variables (hosp0-hosp4) and cLOS variables (los0-los4)** (screenshots from MPlus output)

**
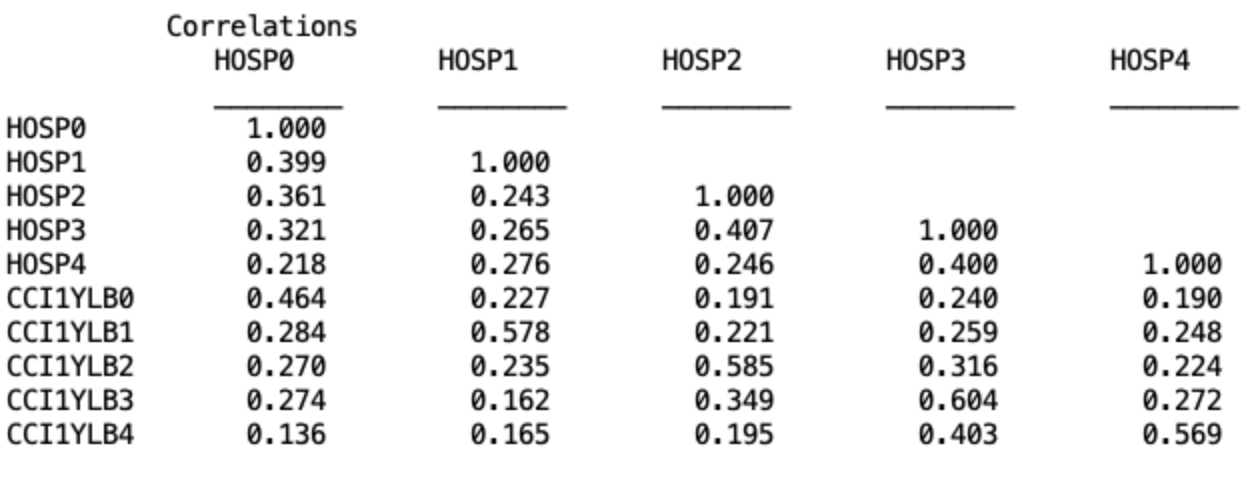
**

**
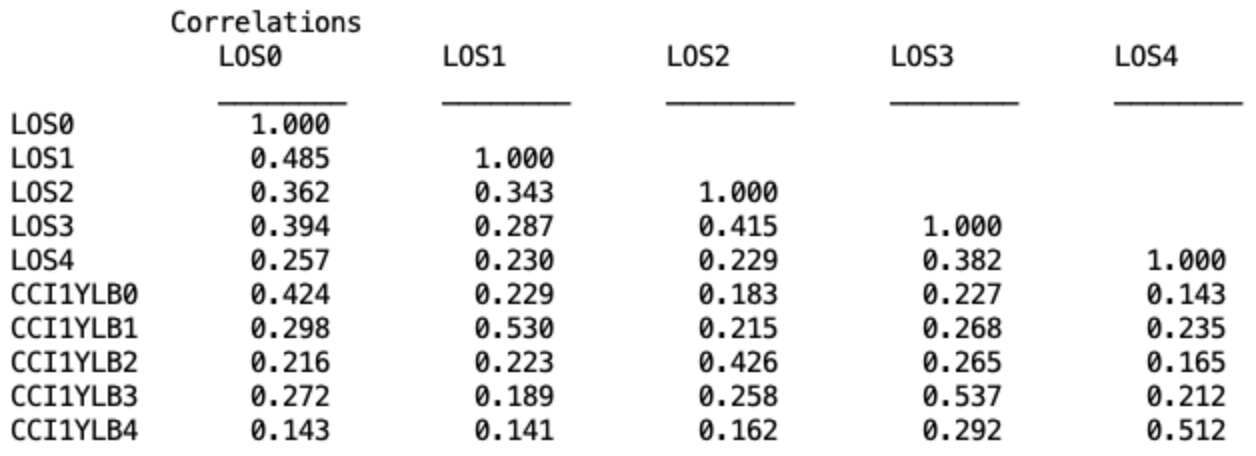
**

# Sydney Memory and Ageing Study Wave 1 Medical History Questionnaire

Now I would like to ask you about medical conditions you have had in the past, or currently have. Remember, all this information is confidential, so please tell me as much as you can.

**Do you have any of the following illnesses at the present time, or have you had them in the past?**

1. Definite Stroke, as diagnosed by a doctor? YES/ NO

*A stroke is characterised by a sudden development of a focal neurological deficit e.g. weakness or paralysis of a limb or one side of the body, numbness or sensory deficits, language difficulties, visual problems (visual field deficit or double vision), loss of balance or articulation difficulties, and considered to be due to disruption of blood supply to the brain.*

If the deficits last > 24 hours, it is a stroke

If the deficits last ≤ 24 hours and recover completely, it is a TIA (mini stroke)

If YES, ask:

1a How many DEFINITE strokes have you had? __________

1b. When was the last stroke? _________________

1c. Which side of the body was affected by the stroke/s? LEFT/ RIGHT

1. TIA (mini stroke) as diagnosed by a doctor? YES/ NO

If YES, ask:

2a. How many DEFINITE TIAs have you had? __________

2b. When was the last TIA? ________

2c. Which side of the body was affected by the TIA? LEFT/ RIGHT

**Have you ever had problems with your heart as diagnosed by a doctor?** YES/ NO

*(If no, prompt a little for problems below, but then skip to Q10)*

1. Heart attack (Myocardial infarction) as diagnosed by a doctor, admitted to hospital and confirmed by electrocardiogram (ECG)? YES/ NO

If YES, ask:

How many heart attacks have you had? _________

1. Angina (chest pain or discomfort on exertion, and sometimes at rest) diagnosed by a doctor? YES/ NO
2. Atrial fibrillation or AF, diagnosed by a doctor? YES/ NO
3. Other cardiac arrhythmia (irregular heart beat) that has needed treatment? YES/ NO
4. Cardiomyopathy? YES/ NO
5. Heart valve disease? YES/ NO
6. Aortic aneurysm? YES/ NO
7. High blood pressure, diagnosed by a doctor? YES/ NO

If YES, ask:

10a. Number of years taken antihypertensive medication? _______

1. High cholesterol, confirmed by a doctor?

If YES, ask:

11a. Number of years taken anticholesterol medication? _______

1. Diabetes, confirmed by a doctor? YES/ NO

If YES, ask:

12a. Age of diagnosis ________ years

12b. How is your diabetes being managed?

- Special diet
- Tablets (oral hypoglycemics)
- Insulin injections

12c. How would you describe your diabetic control?

- Very good
- Satisfactory
- Poor
- Very poor

12d. Have you been told by a doctor that you have had hypoglycemic episodes?

YES/ NO

If YES, ask:

12e. How many? _________

1. Has a doctor ever diagnosed you with circulation trouble or claudication (pain with exercise) in your legs? YES/ NO

**The next few questions ask about head injury.**

1. Have you ever seen a doctor or been to hospital for a head injury? YES/ NO
2. Have you ever had a head injury severe enough to cause loss of consciousness?

YES/ NO

If YES, ask:

15a. How many requiring medical attention? _________

Please now consider your most severe or worst head injury.

15b. For how long did you lose consciousness? ___________________

1. How many general anaesthetics have you had? _______________

**Now I’m going to ask you about other illnesses or disease. Has a doctor ever diagnosed you with:**

1. Migraines? YES/ NO
2. Long standing kidney disease YES/ NO

If YES, ask:

18a. Are you, or have you ever been on dialysis? YES/ NO

1. Urinary Tract Infections (UTIs)? YES/ NO

If YES, ask:

19a. Do you currently have a UTI? YES/ NO

1. Chronic lung disease?

- Asthma
- Emphysema
- Chronic Obstructive Pulmonary Disease (COPD)
- Chronic bronchitis, or do you cough for at least 4 days a week for more than 2 weeks a year for more than 2 years?

If YES, ask: Do you cough up phlegm? YES/ NO

1. Arthritis? YES/ NO

If YES, ask:

21a. What was the diagnosis?

- Osteoarthritis
- Rheumatoid arthritis
- Other __________________________________
- Gout

1. Anaemia? YES/ NO

If YES, ask:

22a. What type?

- Iron deficiency
- Pernicious anemia
- B12 deficiency
- Haemolytic

1. A vitamin deficiency? YES/ NO

If YES, ask:

23a. What type?

- Low B12
- Low Folate
- Low Iron

1. Thyroid disorder? YES/ NO

If YES, ask:

24a. Was it:

- Overactive
- Underactive
- Don’t know

24b. Has this been treated with surgery, radiation, or prescription medication?

YES/ NO

1. Osteoporosis or brittle bones? YES/ NO
2. An autoimmune disease? YES/ NO

If YES, ask:

26a. Was it:

- Lupus
- Sjogren’s disease
- Scleroderma

1. Have you taken anti-inflammatory agents (non-steroidal)? These are drugs usually taken for arthritis or chronic pain. YES/ NO

If YES, ask:

27a. Which one/s have you taken for 6 mths or more in the past?

- Celebrex (celecoxib)
- Brufen (ibuprofen)
- Dolobid (diflunisal)
- Feldene (piroxicam)
- Indocid (indomethacin)
- Naprosyn (naproxen)
- Nurofen (ibuprofen)
- Orudis (ketoprofen)
- Voltaren (diclofenac)
- Other: _____________________________________________________

1. Have you ever been diagnosed with cancer or leukemia? YES/ NO

If YES, ask:

Where was the cancer? What was the treatment? When was it diagnosed?

- Brain (*Exclude) Surgery/ Chemo/ Radio When: ______________
- Lungs Surgery/ Chemo/ Radio When: ______________
- Breast Surgery/ Chemo/ Radio When: ______________
- Uterus Surgery/ Chemo/ Radio When: ______________
- Ovary Surgery/ Chemo/ Radio When: ______________
- Prostate Surgery/ Chemo/ Radio When: ______________
- Stomach Surgery/ Chemo/ Radio When: ______________
- Colon Surgery/ Chemo/ Radio When: ______________
- Small bowel Surgery/ Chemo/ Radio When: ______________
- Blood Surgery/ Chemo/ Radio When: ______________
- Lymph nodes Surgery/ Chemo/ Radio When: ______________
- Pancreas Surgery/ Chemo/ Radio When: ______________
- Kidney Surgery/ Chemo/ Radio When: ______________
- Liver Surgery/ Chemo/ Radio When: ______________
- Bone Surgery/ Chemo/ Radio When: ______________
- Muscle Surgery/ Chemo/ Radio When: ______________
- Nose/pharynx Surgery/ Chemo/ Radio When: ______________
- Melanoma Surgery/ Chemo/ Radio When: ______________
- BCC 🞎 SCC 🞎 Keratoma
- Any other cancers? _______________________________________

Treatment used: _________________________________________

If malignant, what is the current status?

- Remission for _________ years
- Still active - Exclude (except for localised Prostate or minor skin cancer)

**Do you currently have, or have you ever had, any of the following diseases that may affect the brain.**

1. Parkinson’s disease or related syndromes? YES/ NO

If YES, ask:

29a. When was it first diagnosed? ____________

1. Epilepsy (confirmed by a neurologist)? YES/ NO

If YES, ask:

30a. When was it first diagnosed? ____________

30b. When was your most recent seizure? ___________

1. Brain infection – such as encephalitis or meningitis? YES/ NO
2. A brain abcess? YES/ NO
3. Hydrocephalus (or “water on the brain”)? YES/ NO
4. Any other disorder that affects your brain? YES/ NO

If YES, ask:

34a. What was the diagnosis? _______________________________

34b. What was the treatment? _______________________________

1. When getting up suddenly from a lying position, do you experience faintness, dizziness, lightheadedness, nausea or blackout? YES/ NO
2. Do you feel your balance is:

- Excellent
- Very good
- Good
- Fair
- Poor

#### **How many falls did you have in the past 18mths (where you hit the floor)? _______**

1. If >0, ask: How many falls in the last 18 mths involving broken bones? ________

**The next questions are concerned with your alcohol consumption over the last year.**

1. In the past year, approx how often have you had a drink containing alcohol?

- Not in the last year
- Monthly or less – go to Q41
- 2 to 4 times a month – go to Q41
- 2 to 3 times a week – go to Q41
- 4 - 6 times a week – go to Q41
- daily – go to Q41

1. If not in the last year, ask: have you ever drunk alcohol? YES/ NO

If YES, go to Q45

If NO, go to Q49

1. How many standard drinks do you have on a typical day when you are drinking?

- 1
- 2 – 3
- 4 – 5
- 6 – 7
- 8 or more

1. How often do you have 6 or more standard drinks on one occasion?

- Never
- Less than monthly
- Monthly
- Weekly
- Daily or almost daily

*Only ask the following if participant drinks more than four drinks a day for a man or more than two a day for a woman:*

1. How often during the last year have you been unable to remember what happened the night before because you had been drinking?

- Never
- Less than monthly
- Monthly
- Weekly
- Daily or almost daily

1. Has a relative, friend or a doctor or other health worker been concerned about your drinking or suggested you cut down?

- No
- Yes, but not in the last year
- Yes, during the last year

**The next few questions are concerned with your drinking in the more distant past.**

1. Was there a time when you drank more heavily than now? YES/ NO

If NO, go to Q49

If YES, say: I want you to think about the time when your drinking was at its highest level, and answer the following questions about that time.

1. How often did you have a drink containing alcohol?

- Monthly or less
- 2 to 4 times a month
- 2 to 3 times a week
- 4 - 6 times a week
- daily

1. How many standard drinks did you have on a typical day when you were drinking?

- 1
- 2 – 3
- 4 – 5
- 6 – 7
- 8 or more

1. For how many years did you drink this amount?

- 1
- 2 – 3
- 4 – 5
- 6 – 7
- 8 or more

**I would now like to ask you some questions about smoking (tobacco).**

1. Have you ever smoked tobacco regularly? YES/ NO

If NO, go to Q56

1. How old were you when you started smoking? _________
2. Have you smoked at all over the last month? YES/ NO

If YES, go to Q52

If NO, go to Q54

1. What do/ did you smoke?

- Cigarettes (commercial)
- Cigarettes (rollies)
- Pipes
- Cigars
- Chew Tobacco

1. How often do/ did you smoke?

- At least once a day

How many do you usually smoke in a day? _______

- Less than once a day

How many do you usually smoke in one month? ______

1. At what age did you stop smoking? ________
2. On average, how many cigarettes would you have smoked each day over the time you were smoking? ________

**I would now like to ask you some questions about your mental health.**

1. In your lifetime, have you ever been diagnosed with depression? YES/ NO

If NO, go to Q57

If YES, ask:

56a. What sort of treatment did you receive?

- Therapy with Psychiatrist
- Therapy with Psychologist
- Therapy with GP
- Hospital admission
- Drugs
- ECT
- Other: _______________________

56b. Has your depression been:

- continuous For how many years? ________
- intermittent How many discrete episodes of depression have you had?

_______

*(An episode is at least 2 weeks of daily depression or loss of interest impairing usual function. For discrete episode, at least 2 months of no signs or symptoms of depression since last episode)*

1. Have you ever seen a health professional for any other mental health issue?

YES/ NO

**Now I’m going to ask some questions about your vision and hearing.**

1. Is your vision adequate for all purposes with glasses on? YES/ NO

If YES, go to Q59.

If NO, choose from below:

- Is your vision to the point where you can't see well in dim light, or require large print for reading, or cannot do fine needle work.
- Is your vision to the point where you can't read or watch TV or perform other 'normal' visual activities; but you do not require assistance with walking in regard to vision.
- Is your vision a serious problem (you are practically blind)?

1. Have you ever had any of the following eye problems diagnosed:

- Glaucoma
- Macular Degeneration
- Cataracts
- Other eye problems? _________________________________

1. Is your hearing adequate for all purposes with hearing aids in? YES/ NO

If YES, go to Q61

If NO, choose from below:

- Is your hearing to the point where you can't hear speech in groups or noisy environments.
- Is your hearing to the point where some words are missed in conversation; or phone conversation is difficult; or people must speak up.
- Is your hearing a serious problem (you are virtually deaf)?

1. Has a hearing aid being recommended to you? YES/ NO

If YES, ask:

61a. Do you usually wear a hearing aid? YES/ NO

*The next few questions are for females only – go to Q65 if interviewing a male.*

1. Have you ever had hormone replacement therapy (HRT) after menopause?

YES/ NO

If YES, ask:

62a. How long have you had or did you have HRT? ______ years

1. What age was your menopause (6 months after last period)? _________
2. Did your menopause occur naturally or was it after surgery (hysterectomy)?

Naturally/ Surgically

**Now I’m going to ask about your weight and height.**

65a. What is your weight (estimation)?

_ _ ___ kgs OR _ ___ stones ____ pounds

65b. How much is the heaviest you’ve ever weighed? *Don’t include when pregnant.*

. _ _ ___ kgs OR _ ___ stones ____ pounds

65c. How tall are you?

_ ___ _ cms OR _ ___ feet. ___ _ inches

**Now I would like to ask you some questions to find out about your exposure to pesticides.**

1. Have you ever worked in agriculture (including livestock)? YES/ NO

If NO, go to Q67

If YES, ask:

66a. What type of agriculture have you worked in?

- Beef cattle
- Dairy cattle
- Sheep
- Poultry
- Grain
- Orchards
- Vineyards
- Cotton
- Rice
- Market Garden
- Sugar
- Other: _________________________________

66b. Did you work directly with farm chemicals? YES/ NO

66c. How many years were you exposed to farm chemicals? __________

66d. Which chemicals? ________________________________________

66e. Did you have any reactions to the chemicals? YES/ NO

1. Are you, or have you ever been, a rose gardener? YES/ NO
2. Up until you were 19 did you ever live in a rural area, if so for how many years?

__________

68a. If YES, ask: What was/ is the name of the town: _________________________

**Now I’m going to ask about some things which may affect your sense of smell.**

1. Have you had a cold or respiratory tract infection within the last 3 – 4 weeks?

YES/ NO

1. Have you suffered from chronic sinusitis or any chronic nasal inflammatory condition? YES/ NO
2. Have you had nasal surgery? YES/ NO

71a. If YES, ask: Was there a decrease in your ability to smell after surgery?

YES/ NO

*If YES to any of questions 69 – 71a, note on B-SIT.*

**Now I’m going to ask you some questions about driving.**

1. Do you currently drive? YES/ NO

If YES, go to Q73.

If NO, ask:

72a. Did you ever drive? YES/ NO *If NO, go to Q75.*

72b. If you stopped driving, at what age did you stop? _______

72c. If you stopped driving, why?

- Enforced (Police/ RTA)
- Recommended (Dr)
- Voluntary
- Other: ________________________________

1. Have you been the at-fault driver in any car accidents in the last 18mths? YES/ NO

73a. How many minor accidents (no personal injuries) _______

73b. How many major accidents (involving personal injuries) _______

1. Do you currently have any restrictions on your driving?

- Enforced (Police/ RTA)
- Recommended (Dr)
- Voluntary
- No restrictions
- Other: ________________________________

**Now I’m going to ask you some questions about your sleep habits.**

1. Have you been told you snore?

- No
- Occasionally
- Regularly

1. Do you have excessive sleepiness during the day which interferes with your normal daytime functioning?

- No
- Yes, lightly dozing
- Yes, heavy sleep

1. Have you ever been diagnosed with sleep apnoea by a specialist? YES/ NO

If YES, ask:

77a. What treatment have you used?

- CPAP machine
- Surgery
- Other: ___________________

77b. When was it diagnosed? ______________

1. Have you ever been told that “act out” your dreams, such as reaching out to hit an intruder? YES/ NO

If YES, ask:

78a. At what age did that start? ________

**Now some questions about your opinion of your health.**

1. In general, would you say your health is:

- Poor
- Fair
- Good
- Very Good
- Excellent

1. Compared to one year ago, how would you rate your health in general now?

- Much better now
- Somewhat better now
- About the same
- Somewhat poorer now
- Much poorer now

1. Compared to other people your age how would you rate your health in general now?

- Much better
- Somewhat better
- About the same
- Somewhat poorer
- Much poorer

**Now, some questions about your teeth.**

1. How often do you visit a dentist?

- Every 6 months
- Every 12 months
- Every 2 years
- Every 3 – 5 years
- Haven’t been in 5 years

1. Has your dentist ever remarked that you suffer from any of the following:

- Bleeding gums (gingivitis)
- Loss of bone supporting the teeth (periodontitis)
- Tooth mobility associated with/ related to gum disease
- Loss of teeth due to gum problems

1. Finally, are there any other serious illnesses you have but have not been mentioned?

______________________________________________________________________

______________________________________________________________________

______________________________________________________________________

______________________________________________________________________

______________________________________________________________________

______________________________________________________________________

______________________________________________________________________

**End of Medical History Questionnaire**

**Centre for Health Record Linkage (CHeReL)
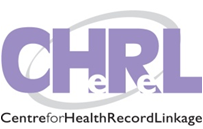
**

**Data Linkage Report 8 December 2015**

**PROJECT: Cognitive decline in the elderly and health service utilisation. (AU RED Ref: HREC/15/CIPHS/11; CI Ref: 2015/03/582; CHeReL Ref: 2014.50-1)**

# METHODS:

### **Sources of data**

- **The Sydney Memory and Aging Study**

The Sydney Memory and Ageing Study (MAS) is a prospective cohort study initiated in 2005 with the primary aim of examining the clinical characteristics and prevalence of mild cognitive impairment in a random sample of non-demented community dwelling older people, and determining the rate of change in cognitive function over time.

- **NSW Admitted Patient Data Collection**

The Admitted Patient Data Collection (APDC) includes records for all hospital separations (discharges, transfers and deaths) from all NSW public and private hospitals and day procedure centres. The APDC records include a range of demographic data items (e.g. date of birth, residential address, language spoken at home and country of birth), administrative items (e.g. admission and separation dates) and coded information (e.g. reason for admission, significant co-morbidities and complications and procedures performed during the admission).

- **NSW Emergency Department Data Collection**

The Emergency Department Data Collection (EDDC) is maintained by the Health System Information and Performance Reporting Branch of the NSW Ministry of Health and provides information about presentations to the Emergency Departments of public hospitals in NSW. The data items included are demographic information, primary diagnosis and other clinical information.

- **NSW Registrar of the NSW Registry of Births, Deaths and Marriages**

The Registrar of the NSW Registry of Births, Deaths and Marriages (RBDM) is required to register all deaths in NSW. When a person dies, the Medical Certificate of Cause of Death is forwarded to the Registry which transcribes the information onto a computer database in un-coded format. A death registration number is assigned to each death.

The NSW Registrar of Births Deaths & Marriages records all births that occur in New South Wales. The Registry uses the details from the birth registration record to produce a NSW Birth Certificate.

- **Australian Bureau of Statistics / Australian Coordinating Registry Cause of Death Unit Record File**

All deaths for which a coronial inquiry is not required must be certified as to cause and date by a registered medical practitioner and the certificate registered by the Registrar of Births, Deaths and Marriages (RBDM) in each State and Territory. Deaths that are referred to a coroner are registered by the coroner at the conclusion of an inquiry into the circumstances of the death. The vast majority of non-coronial deaths are registered with the relevant RBDM within four weeks of the date of death; however coronial inquiries can take months or even years to conclude.

Details of all registered deaths are forwarded to the Australian Bureau of Statistics (ABS). The ABS then check and code the information. A single code for an underlying cause of death was applied in the years to 1997. From 1997 multiple cause of death codes (ICD-10) were applied to each death record where more than one cause contributed to the death.

The Centre for Epidemiology and Evidence, NSW Ministry of Health receives coded cause of death data from the Australian Coordinating Registry (ACR) for the Cause of Death Unit Record File (COD URF). Access to the COD URF is restricted to support the ACR terms and conditions.

The legacy ABS mortality data prior to 2006 have been mapped and combined with COD URF.

**Data Linkage**

***External Datasets***

The Sydney Memory and Aging Study (MAS) data was provided by the custodian with following identifiers for the linkage;

- Surname
- First and middle names
- Alternative surname, first and middle names (where applicable)
- Gender
- Date of birth
- Address
- Suburb
- Postcode

***Master Linkage Key***

Identifying information such as name, address, date of birth and gender for each dataset is included in the Master Linkage Key (MLK). No health/content data are used in this process.

The MLK is being constructed by the Centre for Health Record Linkage (CHeReL)^1^ using probabilistic record linkage methods and *ChoiceMaker* software^2^. ChoiceMaker uses ‘blocking’ and ‘scoring’ to identify definite and possible matches. During blocking, *ChoiceMaker* searches the target datasets for records which are possible matches to each other. There are two types of blocking. The exact blocking algorithm requires records to have the same set of valid fields and the same values for these fields. The automated blocking algorithm builds a set of conditions that are used to find as many as possible records that potentially match each other. Scoring employs a combination of a probabilistic decision, which is computed using a machine learning technique, and absolute rules, which include upper and lower probability cut-offs, to determine whether each potential match denotes or possibly denotes the same person. Upper and lower probability cut-offs initially start at 0.75 and 0.25 for a linkage and are adjusted for each individual linkage to ensure false links are kept to a minimum. At the completion of the process, each record in the MLK is assigned a record identification number and a MLK person ID to allow linked records for the same individual to be identified and extracted.

**Linkage *of APDC, EDDC, and RBDM Death registrations from MLK to MAS***

NSW APDC, NSW EDDC and NSW RBDM Death records were extracted from the MLK (**Version 2015_16**) for the periods shown in Table 1.

The MLK extract comprising records of the NSW APDC, NSW EDDC and NSW RBDM Death records were linked to the MAS data using probabilistic record linking methods and ChoiceMaker software^2^.

***Deterministic linkage of RBDM deaths with COD URF mortality data***

COD URF records do not contain personal identifiers, only death registration and year. Identifiers are obtained by using a deterministic linkage to RBDM deaths matching on the corresponding fields.

A deterministic linkage (i.e., exact matching) of the COD-URF records to the RBDM death records was carried out using the following 5 step standard procedure:

First pass year of registration, encrypted registration number and exact date of death.

Second pass year of registration, encrypted registration number and either:

1 day difference in date of death or

same year of death or

date of birth.

Third pass year of registration with difference of 1 digit, encrypted registration number and date of birth and sex and date of death

Fourth pass year of registration, date of death, sex, postcode and date of birth

Fifth pass date of death, sex and date of birth.

**Final processing**

Once the linkages were finalised, the CHeReL created a Project Person Number (PPN) for each person identified in the linkage, and assigned this PPN to the relevant MAS cohort, APDC, EDDC, RBDM Deaths and COD URF records. The CHeReL returned the PPN and the encrypted record number from the source databases to the data custodians. The data custodians will supply datasets comprising the approved information from the source database plus the PPN to the project investigators. The investigators can then merge the datasets using the PPN.

**RESULTS**

Table 1 shows the total number and type of records from each data source.

Table 2 summarises the outcome of linking the APDC, EDDC, RBDM Death and COD URF records to the MAS records. All records from cohort and linked records to the cohort for all datasets were returned.

The MLK is regularly checked for false positive linkages. The parameters for the extract from the MLK were set such that we are confident that no true matches were missed if full identifiers were available.

False positive rate = 5/1,000 records (0.5%)

**Table 1**: Data sources and record types

| **Data Source** | **Description** | **Number** |
| --- | --- | --- |
| Sydney Memory and Aging Study  **(MAS)** | All persons enrolled in the MAS study | 1,026 records |
| NSW Admitted Patient Data Collection  **(NSW APDC)** | Episodes of care selected for the following parameters:  Admission dates: 1 Jul 2001 to 30 Jun 2014*  Separation dates: 1 Jul 2001 to 30 Jun 2014* | 31,888,594 records |
| NSW Emergency Department Data Collection  **(NSW EDDC)** | Presentations to Emergency Departments  Admission dates: 1 Jan 2005 to 30 Jun 2015 | 22,742,211 records |
| NSW RBDM death registrations  **(RBDM deaths)** | Death registrations selected for the following parameters:  Death date: 1 Jan 2005 to 30 Jun 2015** | 508,294 records |
| Cause of death, unit record file  **(COD URF)** | All COD URF Data in NSW, selected for following parameters;  Death date: 1 Jan 2005 to 31 Dec 2013 | 426,236 records |
| ***** A change to data processing rules for a subset of hospitals’ admitted patient activity was implemented during 2013/14 financial year. While the impact of these changes was found to be negligible for 2013/14 financial year, there has been a temporary reduction in the coverage of admitted patient hospital activity for 2014/15. As this may affect the quality of record linkage and study findings, admitted patient data beyond 30 June 2014 are being temporarily withheld from release, until a solution can be implemented. The Ministry of Health is currently investigating a range of solutions. | | |
| ** NSW Health receives daily feeds of death registrations. This provides data that is as up to date as possible. However as a result of the continuous updates, duplicates may occur when deaths are notified multiple times. Researchers should determine which records to retain for their purposes. | | |

**Table 2:** Summary of records returned to Study Investigators – Mothers component

| **Data Source** | **Record type** | **Number** |
| --- | --- | --- |
| Sydney Memory and Aging Study  (MAS Cohort) | MAS cohort records linked to other datasets | 1,016 records  (1,016 persons)  99.0% |
|  | Unlinked MAS cohort records | 10 records  (10 persons)  1.0% |
|  | **Total MAS records** | **1,026 records**  **(1,026 persons)** |
| NSW APDC | APDC records linked to MAS cohort  Admission date: 1 Jul 2001 to 30 Jun 2014  Separation date: 1 Jul 2001 to 30 Jun 2014 | 16,268 records  (1,009 persons)  98.3% |
| NSW EDDC | EDDC records linked to MAS cohort  Admission date: 1 Jan 2005 to 30 Jun 2015 | 4,349 records  (872 persons)  85.0% |
| NSW RBDM Deaths | RBDM Deaths records linked to MAS cohort  Death date: 1 Jan 2005 to 30 Jun 2015 | 276 records (273 persons)  26.6% |
| COD URF data | COD URF records linked to MAS cohort  Death date: 1 Jan 2005 to 31 Dec 2013 | 208 records*  (208 persons)  20.3% |
| **Total records returned to Study Investigators:**  **Total Project Person Numbers (PPN):** | | **22,127 records**  **(1,026 persons)** |
| ***Note:** There were 64 RBDM records with death date after 31/12/2013 which will not match to COD-URF records. | | |

***References***

1. The Centre for Health Record Linkage at: [www.cherel.org.au](http://www.cherel.org.au).
2. ChoiceMaker Technologies, Inc. New York, NY 10010.

**Additional Files**

- APDC variables selected for analysis p 266

**Additional info on the MAS sample with regards to ABS figures for those geographical areas**

Comparing males and females, more males had tertiary education (61 vs 29% *p* < .001) and more females lived alone (30 vs 60% *p* < .001). More males than females were treated for cholesterol levels but more females had levels greater than 6.5 mmol/L.

**Selection of CNS primary diagnosis admissions**

The following Australian Refined Diagnosis-Related Groups (AR-DRG) codes were selected: B01A B01B B02A B02B B04A B04B B42A B42B B66A B66B B67A B67B B68A B68B B69A B69B B70A B70B B70C B70D B72A B72B B73Z B74A B74B B76A B76B B78A B78B B79A B79B B80A B80B ^17^. Some of the diagnosis codes are ambiguous with regards to specific clinical presentation (e.g. degenerative neurological diseases AR-DRG code B67) and thus, this process may have included some CNS admissions with minimal cognitive impact.

**Expanded version of base model description**

After establishing measurement invariance using a Longitudinal Confirmatory Factor Analysis (LCFA) (Figure S1), a CFM was run without hospitalization predictors to estimate the intercept and slope of global cognition (Figure S2). Factor loading for the latent intercept was set to 1, while those for the latent slope were set to 0, 2, 4 and 6 for Waves 1 to 4 respectively, to reflect the spacing of time. The latent intercept and slope represent the estimated initial level of and linear change in global cognition per year, respectively. To facilitate interpretation of the unstandardised effects, the latent intercept was fixed with a mean of 0 and a variance of 1, such that change in cognition could be interpreted as change in standard deviation against an average person at baseline.

**Difference between self-reported Sydney Memory and Ageing Study comorbidity data and New South Wales Admitted Patient Data Collection (APDC) (electronically-linked) comorbidity data**

Information on comorbidities from APDC data differed from self-report comorbidity data. These differences may arise from discrepancies between the accuracy of comorbidities coded during admission, medical definitions of diagnoses being coded and self-report perceptions of the presence of medical conditions. For the purposes of this analysis, self-report was used for reasons mentioned in section 3.3 and additionally because Charlson Comorbidity Index (CCI) was not coded for the minority of the MAS sample with no hospitalization episodes. For similar studies ^18-20^, demographics and comorbidities were broadly comparable, although the heterogenous nature of comorbidity data collection methods make comparisons challenging. Additional analyses were also added adjusting for CCI.

**Admitted Patient Data Collection (APDC) (electronically linked) available variables (https://www.cherel.org.au/datasets):**

# Variable Information

| Variable | Description/Notes | Name in dataset | Codes |
| --- | --- | --- | --- |
| Hospital type | Flag to indicate if facility is public or private- based on the source system. | hospital_type | See Codes: Hospital Type |
| Facility type | The category of the facility through which the health service is delivered. | facility_type | See Codes: Facility Type |
| Peer group | Facility peer grouping (Public Hospitals only) For more information please see: <https://www1.health.nsw.gov.au/pds/Pages/doc.aspx?dn=IB2016_013> | peer_group | See Codes: Peer Group |
| Acute Hospital Flag | Indicates whether or not the patient received the service at an acute facility. | acute_flag |  |
| Recognised public hospital flag | Flag to identify whether the patient attended a facility that was a public hospital recognised by the Commonwealth Government under the Medicare Agreement. | recognised_ph_flag |  |
| Local Health District of Facility |  | area_identifier | See Codes: Local Heath District (LHD) |
| Facility identifier | The specific hospital, nursing home or day procedure centre reporting the inpatient episode of care. Where information on specific facilities is required, specify by name. | facility_identifier_recode | Code lists are updated regularly. |
| Stay number (encrypted) | The stay number for each period of stay [encrypted] | stay_number_e |  |
| Episode sequence number | The sequence number of an episode during a period of stay. | episode_sequence_number |  |
| Episode start date | The date on which an admitted patient commences an episode of care, by either a formal admission to the hospital or a type change to a subsequent episode within the one stay in hospital. Full date will only be supplied if sufficient justification is supplied. Date may otherwise be supplied as year, year and month, or year, month and day of the week only | episode_start_date |  |
| Episode start time | The time at which an admitted patient commences an episode of care, by either a formal admission to the hospital or a type change to a subsequent episode within the one stay in hospital. | episode_start_time |  |
| Episode end date | The date on which an admitted patient completes an episode of care, by either a formal discharge from the hospital or by a statistical type change to a subsequent episode. Full date will only be supplied if sufficient justification is supplied. Date may otherwise be supplied as year, year and month, or year, month and day of the week only | episode_end_date |  |
| Episode end time | The time at which an admitted patient completes an episode of care, by either a formal discharge from the hospital or by a statistical type change to a subsequent episode. | episode_end_time |  |
| Episode day stay length of stay in hours | The number of hours a patient who is admitted and separated on the same day is admitted to the hospital. From 2014-15 only available for public hospitals | episode_day_stay_los_recode. |  |
| Episode leave days total | The total number of days the patient was not at the hospital between the date of admission and separation. Periods of leave may be up to 7 days, however there is no limit to the number of periods of leave a patient can take during an episode of care. A large number of leave days are common for psychiatric patients. | episode_leave_days_total |  |
| Episode length of stay | The number of days the patient spends in the hospital. From 2014-15 only available for public hospitals | episode_length_of_stay | (episode end date –episode start date – leave days) |
| Episode of care type | This item is used to record the principal clinical intent or treatment goal of the care provided to the patient for the episode of care. | episode_of_care_type | See Codes: Episode of Care Type |
| Mode of separation | The method (discharge, death, transfer, etc) by which the patient separates from the episode of care | mode_of_separation_recode | See Codes: Mode of Separation |
| DRG mode of separation | Status at separation of person (discharge / transfer / death) and place to which the person is released. From 2014-15 only available for public hospitals.. | drg_mode_of_separation | <https://meteor.aihw.gov.au/content/index.phtml/itemId/270094> |
| Contract status | An indication whether or not the admitted patient service being provided during this stay in hospital is being performed under a contractual agreement with another facility or health service. (Formerly known as ’contract’) | contract_status_public | See Codes: Contract Status |
|  |  | contract_status_private |  |
| Facility transferred from | The hospital, nursing home or day procedure centre the patient was transferred from. | facility_trans_from_recode |  |
| Facility transferred to | The hospital, nursing home or day procedure centre the patient was transferred to. | facility_trans_to_recode |  |
| Emergency status | Urgency of admission. Indicates whether or not, in the opinion of the treating clinician, the admission was an emergency: that is, care or treatment was required within 24 hours. Applies to public hospital data only | emergency_status_recode | See Codes: Emergency Status |
| Emergency Department Status | A flag that indicates whether a patient during an episode of care has been treated within the emergency department, and if so, whether they were also admitted to a ward. | ed_status | See Codes: ED Status |
| Referred to on separation | This variable records the service to which the patient was referred on separation from this episode of care (re-coded by adding a leading zero to values 1-9) | referred_to_on_separation_recode | See Codes: Referred to on Separation |
| Source of referral | The source from which the person was referred to the hospital (re-coded, by adding a leading zero to values 0-9). Refer to data custodian for codes for historical years of data | source_of_referral_recode | See Codes: Source of Referral |
| Unit type on admission | The designation of each bed, in terms of type of care or group of patients, which the patient is accommodated in during his/her stay in hospital. From 2014-15 this variable is only available for public hospitals. | unit_type_on_admission | See Codes: Unit Type on Admission |
| Hours in ICU | The number of hours the patient spent in a designated intensive care unit for this episode of care. | hours_in_icu |  |
| Hours on mechanical ventilation | The total number of completed hours that the patient has spent on mechanical ventilation during the episode of care. | hours_on_mech_ventilation |  |
| Involuntary days in psychiatric unit | The sum of the number of days or part days of the episode of care that the person was an involuntary patient under the Mental Health Act, minus the sum of leave days occurring during the episode within the designated unit. | involuntary_days_in_psych |  |
| Last psychiatric admission date | Where the person has had a previous admission to a designated psychiatric unit in any facility, the year that the person was last separated from the designated psychiatric unit. From 2014-15 only available for public hospitals. Full date will only be supplied if sufficient justification is supplied. Date may otherwise be supplied as year, year and month, or year, month and day of the week only | last_psych_admission_date |  |
| Days in Psychiatric Unit | If a patient has been admitted to a designated psychiatric unit at any time during the episode of care, enter the number of days the patient was accommodated in the designated psychiatric unit. | days_in_psych_unit |  |
| Qualified bed days | Used in the calculation of Unqualified/Qualified Baby Bed Days. | qualified_bed_days_recode |  |
| Unqualified baby bed days | The number days a newborn was unqualified under the Health Insurance Act. | unqual_baby_bed_days |  |
| Australian Refined Diagnosis Related Group | From 2014-15 ARDRG is available for public hospitals only | ar_drg | <https://www.ihpa.gov.au/admitted-acute-care/ar-drg-classification-system> |
|  | Version number of the ARDRG codeset (essential for interpreting ar_drg) | ar_drg_version |  |
| Major Diagnostic Category | Major Diagnostic Category (MDC) for the ARDRG | MDC | See Codes: Major Diagnostic Category |
| Service Related Group | Classifies patients according to the type of speciality service they principally receive. From 2014-15 only available for public hospitals | SRG | See Codes: Service Related Groups |
|  | SRG version (essential for interpreting SRG) | SRG_version |  |
| Clinical codeset | The classification scheme used to code a procedure or diagnosis (clinical_codeset is essential for interpreting diagnosis and procedure codes) | clinical_codeset |  |
| Condition onset flags | A qualifier for each coded diagnosis indicating the onset of the condition relative to the beginning of the episode of care.. Available from 1 July 2008 | condition_onset_flagP, condition_onset_flag1-condition_onset_flag50 | See Codes: Condition Onset |
| Diagnosis codes | Diagnoses for the episode of care coded according to ICD-10-AM edition current at the end of the episode. Principal diagnosis has ‘P’ suffix | diagnosis_codeP, diagnosis_code1-diagnosis_code50 | ICD-10-AM |
| Procedure codes | The ACHI code specific to each procedure undertaken during an episode of care, defining what was performed during the associated episode/event. Principal (or first listed) procedure has ‘P’ suffix. | procedure_codeP, procedure_code1-procedure_code50 | ICD-10-AM ACHI |
| Procedure block number | The ACHI block specific to each procedure code | procedure_blockP, procedure_block1-procedure_block50 | ICD-10-AM ACHI |
| Procedure location | The location where the procedure was delivered. | procedure_locationP, procedure_location1-procedure_location49 |  |
| Health insurance on admission | Indicates whether the person receiving the inpatient service is insured with top cover or basic cover, or not insured at the time of admission. This variable is not intended to indicate whether or not the person utilises hospital benefit entitlements. | health_insurance_on_admit | See Codes: Health Insurance on Admission |
| Payment status on separation | Indicates the payment status of the patient. | payment_status_on_sep | See Codes: Payment Status on Separation |
| Department of Veterans Affairs card type | Indicates the type of Veterans Affairs card | DVA_card_type | 1 =White Card; 2 = Gold Card; 3 = Orange Card |
| Financial class | This information should be determined by the hospital based on the patient’s Medicare eligibility, election to be treated by a hospital or hospital doctor, election of single or private room accommodation, compensable status, DVA status, same day/overnight status, etc. This variable is available for public hospitals only. | financial_class | See Codes: Financial class |
| Financial program | The code used to represent the financial program recorded for an episode of care to indicate the type of service under which the episode was categorised. This variable is available for public hospitals only. | financial_program | See Codes: Financial Program |
| Financial sub program | A code to identify the various components of the mental health financial program to aid in the identification of service specific activities, and where the primary episode of care costs were incurred. Available for public hospitals only. | financial_sub_program | See Codes: Financial Sub-program |
| Sex | The biological sex of the patient. | sex | See Codes: Sex |
| Age | The age in years of the patient (derived) | age_recode | (episode start date – date of birth) |
| Age group | Five year age group, derived from re-coded age | age_grouping_recode | See Codes: Age Group |
| Birth date | Full date of birth will only be supplied if sufficient justification is supplied that age in years is insufficient. Year and month may be provided | birth_date |  |
| Country of birth (SACC) | Coding for this variable in private data may not be according to the SACC 2016 | country_of_birth_SACC | [SACC 2016](https://www.abs.gov.au/ausstats/abs@.nsf/mf/1269.0) |
| Country of usual residence | Coding for this variable in private data may not be according to the SACC 2^nd^ Edition | country_of_usual_residence | [SACC 2^nd^ edition](https://www.abs.gov.au/AUSSTATS/abs@.nsf/Lookup/1269.0Main+Features1Second%20Edition) |
| Marital status | The marital status of the patient on admission to the episode of care | marital_status | See Codes: Marital Status |
| Indigenous status | Whether the person is Aboriginal or Torres Strait Islander, based on the person’s own self-report. See notes above regarding access to this variable. | indigenous_status | See Codes: Indigenous Status |
| State of residence | Indicates the Australian state of residence for the patient. | state_of_recidence_recode | See Codes: State of Residence |
| LHD of residence | Local Health District of residence (2010 boundaries) | LHD_2010_code | See Codes: Local Heath District (LHD) |
| Primary Health Network 2015 | Primary Health Network 2015 | PHN_2015_Code | [PHN Boundaries](https://www1.health.gov.au/internet/main/publishing.nsf/Content/PHN-Boundaries) |
| Australian Statistical Geography Classification (ASGC) 2001 Boundaries | Statistical Local Area 2001 | SLA_2001_code | [Australian Statistical Geography Classification 2001](mailto:•%09https://www.abs.gov.au/AUSSTATS/abs@.nsf/Lookup/1216.0Main+Features12001) |
|  | Local Government Area 2001 | LGA_2001_code |  |
| Australian Statistical Geography Classification (ASGC) 2006 Boundaries | 2006 Statistical Local Area 2006 | SLA_2006_code | [Australian Statistical Geography Classification 2006](https://www.abs.gov.au/AUSSTATS/abs@.nsf/Lookup/1216.0Main+Features1Jul%202006) |
|  | Local Government Area 2006 | LGA_2006_code |  |
| Australian Statistical Geography Classification (ASGC) 2011 Boundaries | Statistical Local Area 2011 | SLA_2011_code | [Australian Statistical Geography Classification 2011](https://www.abs.gov.au/ausstats/abs@.nsf/PrimaryMainFeatures/1216.0.15.001?OpenDocument) |
| Australian Statistical Geography Standard (ASGS) 2011 Boundaries | Statistical Area Level 2 | SA2_2011_code | [Australian Statistical Geography Standard 2011](mailto:•%09https://www.abs.gov.au/AUSSTATS/abs@.nsf/Lookup/1270.0.55.001Main+Features1July%20201) |
|  | Statistical Area Level 3 | SA3_2011_code |  |
|  | Statistical Area Level 4 | SA4_2011_code |  |
|  | Local Government Area 2011 | LGA_2011_code |  |
| Australian Statistical Geography Standard (ASGS) 2016 Boundaries | Statistical Area Level 2 | SA2_2016_code | [Australian Statistical Geography Standard 2016](https://www.abs.gov.au/ausstats/abs@.nsf/mf/1270.0.55.001) |
|  | Statistical Area Level 3 | SA3_2016_code |  |
|  | Statistical Area Level 4 | SA4_2016_code |  |
|  | Local Government Area 2016 | LGA_2016_code |  |

**References**

1. CHeReL. Master Linkage Key Quality Assurance. 2012;

2. Sachdev PS, Brodaty H, Reppermund S, et al. The Sydney Memory and Ageing Study (MAS): methodology and baseline medical and neuropsychiatric characteristics of an elderly epidemiological non-demented cohort of Australians aged 70-90 years. Research Support, Non-U.S. Gov't. *International Psychogeriatrics*. Dec 2010;22(8):1248-1264. doi:<http://dx.doi.org/10.1017/S1041610210001067>

3. McLennan W. *ASCO: Australian Standard Classification of Occupations*. 1997.

4. Nelson HE, Willison, J. *National Adult Reading Test (NART): Test Manual*. 2nd Edition ed. NFER Nelson; 1991.

5. Wechsler D. *Wechsler Adult Intelligence Scale-III*. The Psychological Corporation; 1997a.

6. Reitan RM, Wolfson, D. *The Halstead-Reitan Neuropsychological Test Battery: Theory and Clinical Interpretation*. 2nd Edition ed. Neuropsychology Press; 1993.

7. Wechsler D. *Wechsler Memory Scale-III manual*. Harcourt Brace & Company; 1997.

8. Rey A. *L’Examen Clinique en Psychologie*. Presses Universitaires de France; 1964.

9. Benton AL, Sivan, A. B. and Spreen, O. *Der Benton Test* 7th edition ed. Huber; 1966.

10. Kaplan E, Goodglass, H. and Weintraub, S. . *The Boston Naming Test*. Lippincott, Williams & Wilkins; 2001.

11. Spreen OaB, A. L. . *Neurosensory Centre Comprehensive Examination for Aphasia Manual (NCCEA)*. University of Victoria; 1969.

12. Wechsler D. *WAIS-R Manual*. The Psychological Corporation; 1981.

13. Benton AL. Problems of test construction in the field of aphasia. *Cortex*. 1967;3:32-58.

14. Klove H. Clinical Neuropsychology. In: Forster FM, ed. *The Medical Clinics of North America*. Saunders; 1963:pp 1647-1658.

15. *An Online Interface for Drawing Path Diagrams for Structural Equation Modeling*. 2016. <http://semdiag.psychstat.org/>

16. Zhang ZY, Ke-Hai. Practical Statistical Power Analysis

using WebPower and R. ISDSA Press; 2018:chap Chaper 19 Drawing Path Diagrams.

17. Australian Institute of Health and Welfare. Data from: Australian refined diagnosis-related groups (AR-DRG) data cubes. Cat. no. WEB 216. <https://www.aihw.gov.au/reports/hospitals/ar-drg-data-cubes> 2019. *Canberra*.

18. Ehlenbach WJ, Hough CL, Crane PK, et al. Association between acute care and critical illness hospitalization and cognitive function in older adults. Research Support, N.I.H., Extramural

Research Support, Non-U.S. Gov't

Research Support, U.S. Gov't, Non-P.H.S. *JAMA*. Feb 24 2010;303(8):763-770.

19. Wilson RSP, Hebert LES, Scherr PASP, et al. Cognitive decline after hospitalization in a community population of older persons. *Neurology*. 2012;78(13):950-956.

20. Brown CHIVMDMHS, Sharrett ARMDP, Coresh JMDPMHS, et al. Association of hospitalization with long-term cognitive and brain MRI changes in the ARIC cohort. *Neurology*. 2015;84(14):1443-1453.

**Centre for Health Record Linkage (CHeReL)
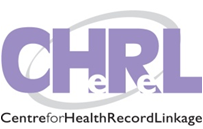
**

**Data Linkage Report 8 December 2015**

**PROJECT: Cognitive decline in the elderly and health service utilisation. (AU RED Ref: HREC/15/CIPHS/11; CI Ref: 2015/03/582; CHeReL Ref: 2014.50-1)**

# METHODS:

### **Sources of data**

- **The Sydney Memory and Aging Study**

The Sydney Memory and Ageing Study (MAS) is a prospective cohort study initiated in 2005 with the primary aim of examining the clinical characteristics and prevalence of mild cognitive impairment in a random sample of non-demented community dwelling older people, and determining the rate of change in cognitive function over time.

- **NSW Admitted Patient Data Collection**

The Admitted Patient Data Collection (APDC) includes records for all hospital separations (discharges, transfers and deaths) from all NSW public and private hospitals and day procedure centres. The APDC records include a range of demographic data items (e.g. date of birth, residential address, language spoken at home and country of birth), administrative items (e.g. admission and separation dates) and coded information (e.g. reason for admission, significant co-morbidities and complications and procedures performed during the admission).

- **NSW Emergency Department Data Collection**

The Emergency Department Data Collection (EDDC) is maintained by the Health System Information and Performance Reporting Branch of the NSW Ministry of Health and provides information about presentations to the Emergency Departments of public hospitals in NSW. The data items included are demographic information, primary diagnosis and other clinical information.

- **NSW Registrar of the NSW Registry of Births, Deaths and Marriages**

The Registrar of the NSW Registry of Births, Deaths and Marriages (RBDM) is required to register all deaths in NSW. When a person dies, the Medical Certificate of Cause of Death is forwarded to the Registry which transcribes the information onto a computer database in un-coded format. A death registration number is assigned to each death.

The NSW Registrar of Births Deaths & Marriages records all births that occur in New South Wales. The Registry uses the details from the birth registration record to produce a NSW Birth Certificate.

- **Australian Bureau of Statistics / Australian Coordinating Registry Cause of Death Unit Record File**

All deaths for which a coronial inquiry is not required must be certified as to cause and date by a registered medical practitioner and the certificate registered by the Registrar of Births, Deaths and Marriages (RBDM) in each State and Territory. Deaths that are referred to a coroner are registered by the coroner at the conclusion of an inquiry into the circumstances of the death. The vast majority of non-coronial deaths are registered with the relevant RBDM within four weeks of the date of death; however coronial inquiries can take months or even years to conclude.

Details of all registered deaths are forwarded to the Australian Bureau of Statistics (ABS). The ABS then check and code the information. A single code for an underlying cause of death was applied in the years to 1997. From 1997 multiple cause of death codes (ICD-10) were applied to each death record where more than one cause contributed to the death.

The Centre for Epidemiology and Evidence, NSW Ministry of Health receives coded cause of death data from the Australian Coordinating Registry (ACR) for the Cause of Death Unit Record File (COD URF). Access to the COD URF is restricted to support the ACR terms and conditions.

The legacy ABS mortality data prior to 2006 have been mapped and combined with COD URF.

**Data Linkage**

***External Datasets***

The Sydney Memory and Aging Study (MAS) data was provided by the custodian with following identifiers for the linkage;

- Surname
- First and middle names
- Alternative surname, first and middle names (where applicable)
- Gender
- Date of birth
- Address
- Suburb
- Postcode

***Master Linkage Key***

Identifying information such as name, address, date of birth and gender for each dataset is included in the Master Linkage Key (MLK). No health/content data are used in this process.

The MLK is being constructed by the Centre for Health Record Linkage (CHeReL)^1^ using probabilistic record linkage methods and *ChoiceMaker* software^2^. ChoiceMaker uses ‘blocking’ and ‘scoring’ to identify definite and possible matches. During blocking, *ChoiceMaker* searches the target datasets for records which are possible matches to each other. There are two types of blocking. The exact blocking algorithm requires records to have the same set of valid fields and the same values for these fields. The automated blocking algorithm builds a set of conditions that are used to find as many as possible records that potentially match each other. Scoring employs a combination of a probabilistic decision, which is computed using a machine learning technique, and absolute rules, which include upper and lower probability cut-offs, to determine whether each potential match denotes or possibly denotes the same person. Upper and lower probability cut-offs initially start at 0.75 and 0.25 for a linkage and are adjusted for each individual linkage to ensure false links are kept to a minimum. At the completion of the process, each record in the MLK is assigned a record identification number and a MLK person ID to allow linked records for the same individual to be identified and extracted.

**Linkage *of APDC, EDDC, and RBDM Death registrations from MLK to MAS***

NSW APDC, NSW EDDC and NSW RBDM Death records were extracted from the MLK (**Version 2015_16**) for the periods shown in Table 1.

The MLK extract comprising records of the NSW APDC, NSW EDDC and NSW RBDM Death records were linked to the MAS data using probabilistic record linking methods and ChoiceMaker software^2^.

***Deterministic linkage of RBDM deaths with COD URF mortality data***

COD URF records do not contain personal identifiers, only death registration and year. Identifiers are obtained by using a deterministic linkage to RBDM deaths matching on the corresponding fields.

A deterministic linkage (i.e., exact matching) of the COD-URF records to the RBDM death records was carried out using the following 5 step standard procedure:

First pass year of registration, encrypted registration number and exact date of death.

Second pass year of registration, encrypted registration number and either:

1 day difference in date of death or

same year of death or

date of birth.

Third pass year of registration with difference of 1 digit, encrypted registration number and date of birth and sex and date of death

Fourth pass year of registration, date of death, sex, postcode and date of birth

Fifth pass date of death, sex and date of birth.

**Final processing**

Once the linkages were finalised, the CHeReL created a Project Person Number (PPN) for each person identified in the linkage, and assigned this PPN to the relevant MAS cohort, APDC, EDDC, RBDM Deaths and COD URF records. The CHeReL returned the PPN and the encrypted record number from the source databases to the data custodians. The data custodians will supply datasets comprising the approved information from the source database plus the PPN to the project investigators. The investigators can then merge the datasets using the PPN.

**RESULTS**

Table 1 shows the total number and type of records from each data source.

Table 2 summarises the outcome of linking the APDC, EDDC, RBDM Death and COD URF records to the MAS records. All records from cohort and linked records to the cohort for all datasets were returned.

The MLK is regularly checked for false positive linkages. The parameters for the extract from the MLK were set such that we are confident that no true matches were missed if full identifiers were available.

False positive rate = 5/1,000 records (0.5%)

**Table 1**: Data sources and record types

| **Data Source** | **Description** | **Number** |
| --- | --- | --- |
| Sydney Memory and Aging Study  **(MAS)** | All persons enrolled in the MAS study | 1,026 records |
| NSW Admitted Patient Data Collection  **(NSW APDC)** | Episodes of care selected for the following parameters:  Admission dates: 1 Jul 2001 to 30 Jun 2014*  Separation dates: 1 Jul 2001 to 30 Jun 2014* | 31,888,594 records |
| NSW Emergency Department Data Collection  **(NSW EDDC)** | Presentations to Emergency Departments  Admission dates: 1 Jan 2005 to 30 Jun 2015 | 22,742,211 records |
| NSW RBDM death registrations  **(RBDM deaths)** | Death registrations selected for the following parameters:  Death date: 1 Jan 2005 to 30 Jun 2015** | 508,294 records |
| Cause of death, unit record file  **(COD URF)** | All COD URF Data in NSW, selected for following parameters;  Death date: 1 Jan 2005 to 31 Dec 2013 | 426,236 records |
| ***** A change to data processing rules for a subset of hospitals’ admitted patient activity was implemented during 2013/14 financial year. While the impact of these changes was found to be negligible for 2013/14 financial year, there has been a temporary reduction in the coverage of admitted patient hospital activity for 2014/15. As this may affect the quality of record linkage and study findings, admitted patient data beyond 30 June 2014 are being temporarily withheld from release, until a solution can be implemented. The Ministry of Health is currently investigating a range of solutions. | | |
| ** NSW Health receives daily feeds of death registrations. This provides data that is as up to date as possible. However as a result of the continuous updates, duplicates may occur when deaths are notified multiple times. Researchers should determine which records to retain for their purposes. | | |

**Table 2:** Summary of records returned to Study Investigators – Mothers component

| **Data Source** | **Record type** | **Number** |
| --- | --- | --- |
| Sydney Memory and Aging Study  (MAS Cohort) | MAS cohort records linked to other datasets | 1,016 records  (1,016 persons)  99.0% |
|  | Unlinked MAS cohort records | 10 records  (10 persons)  1.0% |
|  | **Total MAS records** | **1,026 records**  **(1,026 persons)** |
| NSW APDC | APDC records linked to MAS cohort  Admission date: 1 Jul 2001 to 30 Jun 2014  Separation date: 1 Jul 2001 to 30 Jun 2014 | 16,268 records  (1,009 persons)  98.3% |
| NSW EDDC | EDDC records linked to MAS cohort  Admission date: 1 Jan 2005 to 30 Jun 2015 | 4,349 records  (872 persons)  85.0% |
| NSW RBDM Deaths | RBDM Deaths records linked to MAS cohort  Death date: 1 Jan 2005 to 30 Jun 2015 | 276 records (273 persons)  26.6% |
| COD URF data | COD URF records linked to MAS cohort  Death date: 1 Jan 2005 to 31 Dec 2013 | 208 records*  (208 persons)  20.3% |
| **Total records returned to Study Investigators:**  **Total Project Person Numbers (PPN):** | | **22,127 records**  **(1,026 persons)** |
| ***Note:** There were 64 RBDM records with death date after 31/12/2013 which will not match to COD-URF records. | | |

***References***

1. The Centre for Health Record Linkage at: [www.cherel.org.au](http://www.cherel.org.au).
2. ChoiceMaker Technologies, Inc. New York, NY 10010.

1. terminology from the Australian Qualifications Framework (AQF) 3. McLennan W. *ASCO: Australian Standard Classification of Occupations*. 1997. [↑](#footnote-ref-1)
2. % impaired on Instrumental Activities of Daily Living: impairment being ≥ 3 on the Bayer-Activities of Daily Living Scale [↑](#footnote-ref-2)
3. short informant questionnaire on cognitive decline in the elderly IQCODE > 3 designated as impairment [↑](#footnote-ref-3)
4. Excludes non-English speaking background (NESB) participants and those with inadequate data to classify as MCI [↑](#footnote-ref-4)
5. Extraction method: Principal Axis Factoring, Rotation method: Oblimin with Kaiser Normalization, rotation converged to 9 iterations [↑](#footnote-ref-5)
6. Independent samples t-test to compare means for males and females, equal variances not assumed [↑](#footnote-ref-6)
7. Three factors standardised against the whole sample at Wave 1 [↑](#footnote-ref-7)
8. Covariances between measured variables (Memory, Language and Executive/Visuospatial function) were included in the model but are not shown for simplicity. [↑](#footnote-ref-8)
9. Covariances between measured variables (Memory, Language and Executive/Visuospatial function) were included in the model but are not shown for simplicity. [↑](#footnote-ref-9)
10. Autoregressive paths between global cognition at each time point are specified such that Global Cognition W2, W3, and W4 capture revisualized change between time points. [↑](#footnote-ref-10)
11. χ^2^ (*df)*: Chi-Square test with degrees of freedom, CFI: comparative fit index, RMSEA: Root Mean Square Error of Approximation, SRMR: Standardised Root Mean Square Residual [↑](#footnote-ref-11)
12. ΔCFI < 0.01 indicates measurement invariance [↑](#footnote-ref-12)
13. The latent global cognition slope and intercept are estimated by the model using full information maximum likelihood for missing scores and unadjusted [↑](#footnote-ref-13)
14. Latent intercept mean set to 0 and SD set to 1 by the model [↑](#footnote-ref-14)
15. Cells shaded grey indicate data not publicly available from the Australian Institute of Health and Welfare (AIHW) specifically for Australians aged over 70, including data for individuals to calculate standard deviations for length of stay and rate of hospitalization [↑](#footnote-ref-15)
16. With relation to timing of cognitive assessments: 87% or 2916 of 3352 were at least 120 days prior to the next cognitive assessment and 74.4% or 2494 of 3352 at least 240 days prior to the next cognitive assessment [↑](#footnote-ref-16)
17. Calculated from hospitalization data using a one-year lookback and the Quan coding algorithm: Quan H, Li B, Couris CM, Fushimi K *et al*. Updating and validating the Charlson comorbidity index and score for risk adjustment in hospital discharge abstracts using data from 6 countries. Am J Epidemiol. 2011;173(6):676-82. [↑](#footnote-ref-17)
18. This variable had a large proportion of missing values (54%) [↑](#footnote-ref-18)
19. Independent samples t-test to compare means for males and females, equal variances not assumed [↑](#footnote-ref-19)
20. Time interval from two years prior to Wave 1 assessment back to first date of APDC data commencing in 2001 (i.e. 2-4 years prior to Wave 1). [↑](#footnote-ref-20)
21. Italicised names are the corresponding hospitalization predictor variables names used in the MPlus syntax and figures 1, 2 and S3i. [↑](#footnote-ref-21)
22. For brevity β values for effects are not shown. [↑](#footnote-ref-22)
23. This is the effect on GCOG2 after controlling for GCOG1, GCOG3 after controlling for GCOG2 and GCOG4 after controlling for GCOG3, respectively i.e. the effect of hospitalization predictors on the change in cognition between time points [↑](#footnote-ref-23)
24. This is the effect on GCOG2 after controlling for GCOG1, GCOG3 after controlling for GCOG2 and GCOG4 after controlling for GCOG3, respectively i.e. the effect of hospitalization predictors on the change in cognition between time points [↑](#footnote-ref-24)
25. *p* < .05 [↑](#footnote-ref-25)
26. Simple effect at lower covariate value (-1 SD) and next column at higher covariate value (+1 SD) [↑](#footnote-ref-26)
27. The maximum Charlson Comorbidity Index with one-year lookback in the relevant time interval was used. For models using intercept and slope cognition outcomes, CCI in pre-Wave 1 (to predict intercept) and pre-Wave 1 and 4 (to predict slope) were used since CCI is cumulative and therefore significant overlap would exist between adjacent intervals. For lagged models the CCI in the preceding interval was used (CCI0 was omitted). [↑](#footnote-ref-27)
28. Age, sex and education effects similar to previous and not shown for brevity [↑](#footnote-ref-28)
29. Age, sex and education effects similar to previous lagged models and not shown [↑](#footnote-ref-29)
30. Age, sex and education effects similar to previous lagged models and not shown [↑](#footnote-ref-30)
31. Simple effect at lower covariate value (-1 SD) and next column at higher covariate value (+1 SD) [↑](#footnote-ref-31)
32. Simple effect at lower covariate value (-1 SD) and next column at higher covariate value (+1 SD) [↑](#footnote-ref-32)
